# Fluorinated Analogs of Organosulfur Compounds from Garlic (*Allium sativum*): Synthesis and Chemistry

**DOI:** 10.3390/molecules30132841

**Published:** 2025-07-02

**Authors:** Eric Block, Benjamin Bechand, Sivaji Gundala, Abith Vattekkatte, Kai Wang

**Affiliations:** Department of Chemistry, University at Albany, State University of New York, Albany, NY 12222, USA; benjaminbechand@fas.harvard.edu (B.B.); godchem@gmail.com (S.G.); abith.ramadevan@gmail.com (A.V.); kwang40@its.jnj.com (K.W.)

**Keywords:** garlic, allicin, vinyl dithiins, ajoene, difluoroallicin, trifluoroajoene, 2-fluoroalliin, 1,2-bis(2-fluoro-2-propenyl)disulfane, 2-fluorothioacrolein, 3-fluoro-5,6-dihydro-4*H*-thiopyrans

## Abstract

We report the first syntheses—from commercially available 3-chloro-2-fluoroprop-1-ene (**9**)—of key garlic-derived compounds containing sp^2^-fluorine. We also report synthesis of fluoro-5,6-dihydrothiopyrans by trapping 2-fluorothioacrolein (**15**). Thus, difluoroallicin (**12**, *S*-(2-fluoro-2-propenyl) 2-fluoroprop-2-ene-1-sulfinothioate) is prepared by peracid oxidation of 1,2-bis(2-fluoro-2-propenyl)disulfane (**11**). *S*-2-Fluoro-2-propenyl-l-cysteine (2-fluorodeoxyalliin, **13**), synthesized from cysteine and characterized by X-ray crystallography, is oxidized to its *S*-oxide, 2-fluoroalliin (**22**). The latter, with alliinase-containing powdered fresh garlic, gives a mixture of **12**, allicin (**1**), and isomers of monofluoroallicin (**23**), indicating that **22** serves as a substrate for garlic alliinase. Upon heating, **12** generates transient **15**, which dimerizes giving difluoro vinyl dithiins **6** and **7**. Ethyl acrylate trapping of **15** affords 5- and 6-substituted 3-fluoro-5,6-dihydro-4*H*-thiopyrans (**19** and **20**). In 1,1,1,3,3,3-hexafluoro-2-propanol (HEFP) as solvent, **12** is converted into trifluoroajoene ((*E*,*Z*)-1-(2-fluoro-3-((2-fluoro-2-propenyl)sulfinyl)prop-1-en-1-yl)-2-(2-fluoro-2-propenyl)disulfane; **18**). Liquid sulfur converts **11** to a (CH_2_=CFCH_2_)_2_S_n_ mixture (n = 4–15), characterized by UPLC-(Ag^+^)-coordination ion spray-mass spectrometry.

## 1. Introduction

**Scheme 1:**
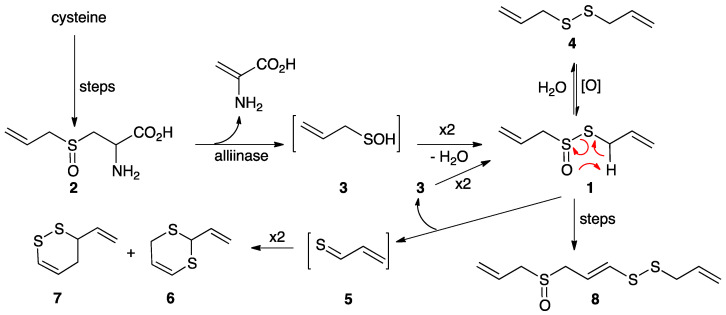
Enzymatic formation of allicin (**1**) from alliin (**2**) by way of 2-propenesulfenic acid (**3**); synthesis of allicin by oxidation of diallyl disulfide (**4**); conversion of **1** to ajoene (**8**); decomposition of **1** to vinyl dithiins (**6** and **7**) via intramolecular elimination to thioacrolein (**5**). In addition to **2**, garlic cloves contain methiin (MeS(O)CH_2_CH(NH_2_)CO_2_H) and isoalliin (MeCH=CHS(O)CH_2_CH(NH_2_)CO_2_H), which give rise to associated structures with methyl and 1-propenyl groups [2,3,4].

Since its discovery by Cavallito and Bailey in 1944 [1], allicin (**1**), the antibiotic active principle of garlic, has been the subject of extensive research [2,3,4,5,6,7,8,9,10]. Enzymatically released from its precursor alliin (**2**) when garlic is crushed, allicin is both unstable and reactive, readily decomposing by way of 2-propenesulfenic acid (**3**) and thioacrolein (**5**) to diallyl disulfide (**4**) and homologous polysulfanes, vinyl 1,2- and 1,3-dithiins (**6** and **7**, respectively) and ajoene (**8**) (Scheme 1). Other thiosulfinates, particularly those with methyl groups, such as MeS(O)SMe and mixed allyl methyl analogs, are also naturally produced, as are more complex compounds [11]. Many of these garlic-derived compounds are biologically active, e.g., as antimicrobial, antiplatelet and anticancer agents [1,2,3,4,5,6,7,8,10,12,13,14,15,16]. In particular, ajoene (**8**) [17,18] has been the subject of several reviews and structure–activity studies due to its notable biological activity as well as its stability, e.g., by Hunter, Kaschula and coworkers [19,20,21,22], Wirth and coworkers [23], and others [24,25,26]. Structure–activity studies and reviews of biological activity have appeared for several of the other garlic-derived compounds, including allicin and other thiosulfinates [27,28,29], *S*-allyl cysteine [30,31,32], disulfides and thiosulfonates [33].

Given the great importance of fluorine in medicinal chemistry and chemical biology [34,35], we were interested in the effect of fluorine substitution for one or more sp^2^-hydrogen atoms in compounds **1**–**8** on the chemical reactivity and biological activity of these compounds. With the exception of CF_3_S(O)SCF_3_, CHF_2_S(O)SCF_3_, and CH_2_FS(O)SCF_3_, whose synthesis but not biological activity was reported [36,37,38], fluorinated analogs of garlic-derived organosulfur compounds are unknown. In line with the current interest in the biological activity of the allyl group [39], our initial goal was to replace one or more vinyl (sp^2^) hydrogens with fluorine, retaining the sp^3^ hydrogens to participate in key intramolecular elimination processes. Compounds with sp^2^ C–F bonds offer advantages associated with the small size, high electronegativity, and metabolic stability of fluorine as well as the altered molecular lipophilicity, membrane fluidity, binding affinity, enhanced volatility, and possibilities for halogen bonding [40] as well as conformational preferences of the fluorinated compounds compared to their hydrogen analogs. Reactivity toward thiol groups, a defining feature of the biological activity of allicin and other garlic compounds [41,42,43], should be enhanced by vinylic fluorine in the thioallylic groups in garlic compounds.

**Scheme 2:**
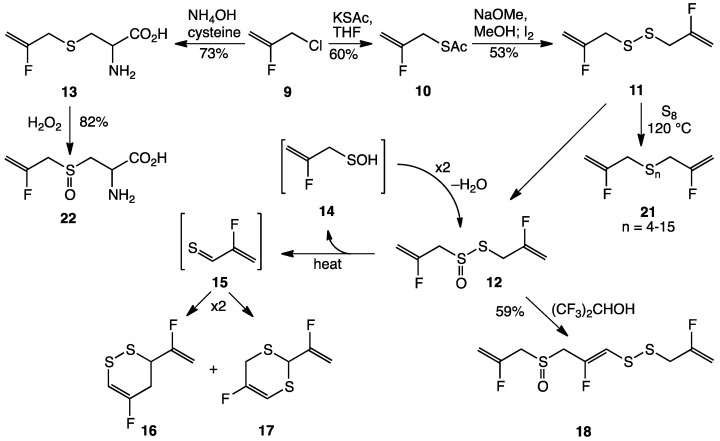
Synthesis of difluoroallicin (**12**) from 3-chloro-2-fluoroprop-1-ene (**9**) by way of thioacetate **10** and bis(2-fluoro-2-propenyl) disulfide (**11**); synthesis of *S*-2-fluoro-2-propenyl-l-cysteine (**13**) from **9**; conversion of **12** to vinyl dithiins **16** and **17** via 2-fluorothioacrolein (**15**) with loss of 2-fluoro-2-propenesulfenic acid (**14**); conversion of **12** to trifluoroajoene **18**; conversion of **11** to polysulfane mixture **21;** conversion of **13** to (+)-*S*-2-fluoro-2-propenyl-l-cysteine *S*-oxide (2-fluoroalliin; **22**).

Two types of C–F substituted allyl groups were considered for this study, namely CH_2_=CFCH_2_–X (**A**) and CF_2_=CFCH_2_–X (**B**). We initially chose type **A** compounds based on commercial availability of CH_2_=CFCH_2_Cl (**9**; Scheme 2) [44,45]; other approaches to fluoroallylation are known [46,47,48,49]. Here, we describe our efforts to synthesize and study the reactivity and activity of difluoroallicin **12**, prepared from **9**, as well as related compounds, such as 2-fluorodeoxyalliin (**13**), 1,2-bis(2-fluoro-2-propenyl)disulfane (**11**), 5-fluoro-3-(1-fluorovinyl)-3,4-dihydro-1,2-dithiin (**16**), 5-fluoro-2-(1-fluorovinyl)-4*H*-1,3-dithiin (**17**), trifluoroajoene (**18**), and bis(2-fluoro-2-propenyl)polysulfanes (**21**).

## 2. Results and Discussion

It was anticipated that difluoroallicin **12** could be prepared by oxidation of disulfane **11**, which in turn could be synthesized from commercially available **9**. Thus, **9** was stirred overnight with potassium thioacetate in THF giving *S*-(2-fluoro-2-propenyl)ethanethioate (**10**). Methanolysis of **10** (NaOMe/MeOH) followed by treatment with iodine gave **11**. Treatment of a chloroform solution of **11** with peracetic acid gave difluoroallicin **12** in 42% yield as a yellow liquid with an onion-like odor. When a chloroform solution of **12** was heated in a sealed tube at 80 °C for 4 h, it decomposed, giving a mixture of dithiin (**16**) as the major product and dithiin (**17**) as the minor product.

**Scheme 3:**
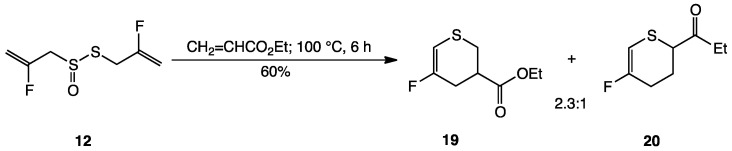
Synthesis of 3-fluoro-5,6-dihydro-4*H*-thiopyran **19** and **20** from difluoroallicin **12**.

Formation of **16** and **17** follows from the proposed mechanism for intramolecular conversion of allicin to thioacrolein and 2-propenesulfenic acid followed by self-Diels-Alder reaction of thioacrolein **5** affording isomeric dithiins **6** and **7** (Scheme 1) [2,3]. To confirm this mechanism, when **12** was heated with a five-fold excess of ethyl acrylate in a sealed tube at 100 °C for 6 h, a 70:30 mixture of the regioisomeric Diels–Alder adducts (**19** and **20**) of ethyl acrylate and **15** was obtained in 60% yield and fully characterized by ^1^H, ^13^C and ^19^F NMR spectroscopy as well as HR-MS (Scheme 3). It is assumed that the second product in decomposition of **12**, 2-fluoro-2-propenesulfenic acid (**14**), self-condenses back to **12** more rapidly than acrylate trapping. While only a single dienophile was used here, it is worth noting that trapping **15** with dienophiles represents a novel and potentially useful synthetic route to 5- and 6-substituted 3-fluoro-5,6-dihydro-4*H*-thiopyrans [50,51]. Thiopyrans display a broad range of biological activity [52] and new synthetic approaches are frequently reported [53,54]. 2-Fluorothioacrolein (**15**) has not been previously reported and represents a novel, reactive heterodiene.

**Scheme 4:**
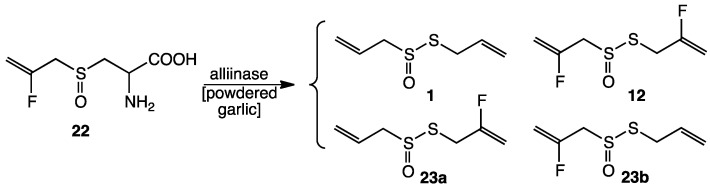
Enzymatic conversion of 2-fluoroalliin **22** to a mixture of **1**, **12** and **23a**,**b**. Alliin, present in the unpurified powdered garlic, provides the C_3_H_5_ group.

**Figure 1:**
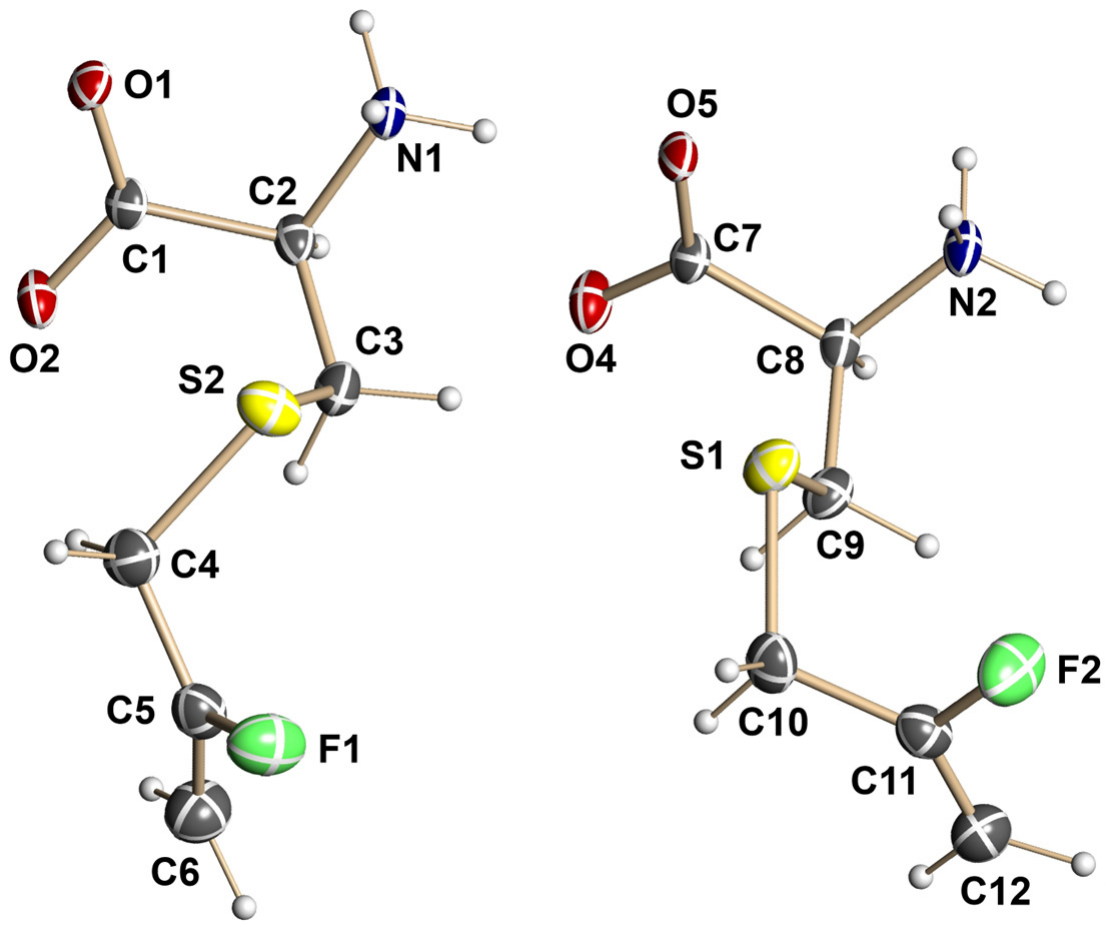
Molecular structure of the l-enantiomer of fluorodeoxyalliin (**13**); non-hydrogen atoms are represented by thermal ellipsoids at the 50% probability level. Color coding: green = fluorine, yellow = sulfur, red = hydrogen, black = carbon.

Treatment of **9** with cysteine in NH_4_OH afforded 2-fluorodeoxyalliin (**13**) in 73% yield. After crystallization, **13** could be characterized by X-ray crystallography as the pure enantiomer shown in Figure 1. Structural data is given in Table 1. Two molecules co-crystallized in the structure; they are just slightly different in bond dimensions. Oxidation of an aqueous solution of **13** (30% H_2_O_2_) gave 2-fluoroalliin (**22**; 82% yield). When **22** was treated with powdered fresh garlic, the presence of **12** and isomers of mono-fluoroallicin **23a**,**b** along with major amounts of allicin **4** was indicated by DART-MS analysis; the presence of **12** and **23a**,**b** was also supported by ^19^F-NMR spectral data (Scheme 4).

When a concentrated solution of **12** in 1,1,1,3,3,3-hexafluoro-2-propanol (HEFP) was kept at room temperature for 16 h, it was converted to a 1:1 mixture of **12** and trifluoroajoene **18** in 59% yield, based on converted **12**. The choice of HEFP follows from the observation that partial conversion of **12** to a mixture of **16**–**18** occurred in isopropanol, and the belief that the higher acidity of HEFP would favor formation of **18**. When the same reaction was conducted at 60 °C, a mixture of **12**, **16** and **18** was formed, suggesting that heat favors formation of **16**. While **18** could be characterized as a mixture with **12** by NMR and liquid chromatography–high resolution mass spectrometry, separation of **12** and **18** was not possible with the chromatographic methods employed, unfortunately precluding biological studies of **18**.

**Scheme 5:**
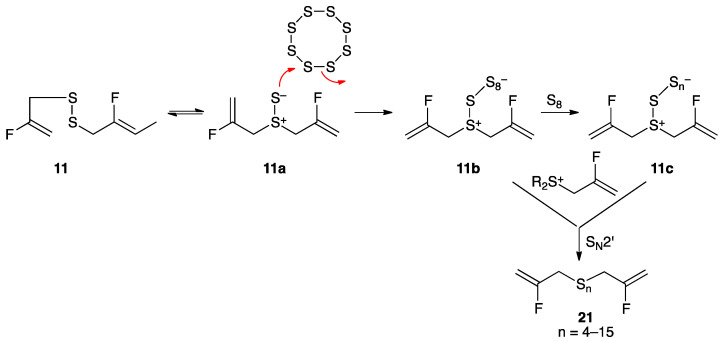
Conversion of disulfide **11** to polysulfane mixture **21** through condensation with molten sulfur. Several equivalents of S_8_ can react, losing different numbers of sulfur atom groupings, followed by S_N_2′ reaction with 2-fluoroallyl sulfonium species giving polysulfane mixture **21**. By other techniques, compounds containing up to 26 sulfur atoms could be observed.

Treatment of disulfide **11** with liquid sulfur at its melting point of 120 °C for 1 h, as described previously for **4** [55], afforded polysulfane mixture **21** with 4–15 sulfur atoms, with compounds containing 4–6 sulfur atoms predominating. A proposed mechanism, initiated by [2,3]-sigmatropic rearrangement of disulfide **11** (Scheme 5) to the dipolar thiosulfoxide isomer, is consistent with computational studies showing that fluorine substitution on the 2-position of the allyl group has little effect on rearrangement to **11a** [56].

**Figure 2:**
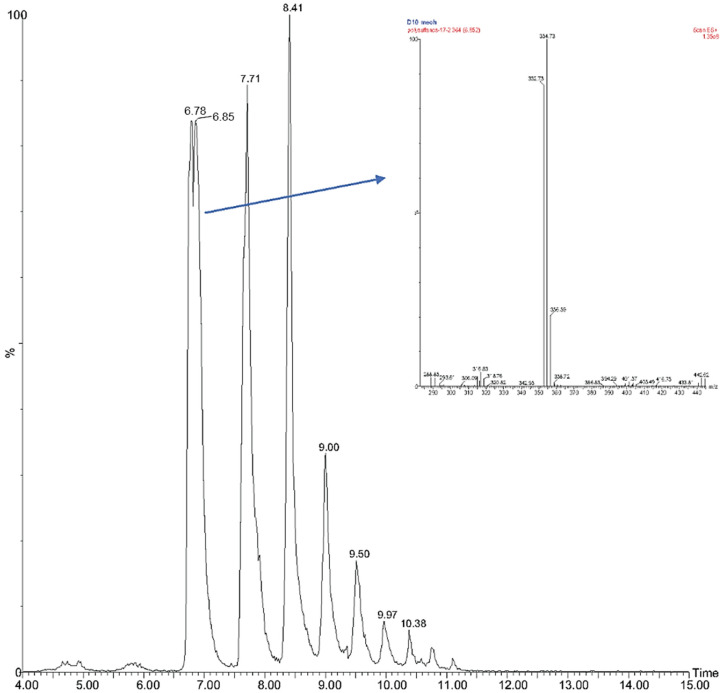
UPLC (Ag^+^)-CIS-MS selective ion chromatogram of polysulfane mixture **21**, with 4 to 15 sulfur atoms: C_6_H_8_F_2_S_4_-^107^Ag complex, *m*/*z* 352.7, 6.78/6.85 min (split peak due to column issues); C_6_H_8_F_2_S_5_-^107^Ag complex, *m*/*z* 384.7, 7.71 min; C_6_H_8_F_2_S_6_-^107^Ag complex, *m*/*z* 416.7, 8.41 min; C_6_H_8_F_2_S_7_-^107^Ag complex, *m*/*z* 448.7, 9.00 min; C_6_H_8_F_2_S_8_-^107^Ag complex, *m*/*z* 480.7, 9.50 min; C_6_H_8_F_2_S_9_-^107^Ag complex, *m*/*z* 512.6, 9.97 min; C_6_H_8_F_2_S_10_-^107^Ag complex, *m*/*z* 544.5, 10.38 min; C_6_H_8_F_2_S_11_-^107^Ag complex, *m*/*z* 576.4, 10.7 min; and C_6_H_8_F_2_S_12_-^107^Ag complex, *m*/*z* 608.4, 11.1 min. With additional signal amplification, other low abundance complexes could also be seen: C_6_H_8_F_2_S_13_-^107^Ag complex, *m*/*z* 640.3, 11.43 min; C_6_H_8_F_2_S_14_-^107^Ag complex, *m*/*z* 672.3, 11.73 min; C_6_H_8_F_2_S_15_-^107^Ag complex, *m*/*z* 704.2, 12.05 min. The insert shows molecular ions *m*/*z* 352.73 and 354.73 corresponding to C_6_H_8_F_2_S_4_-^107^Ag and C_6_H_8_F_2_S_4_-^109^Ag. The chromatogram selecting for C_6_H_8_F_2_S*_n_*-^109^Ag complexes was very similar.

Because weak signals were obtained using standard LC-CIS-MS techniques, analysis of the complex polysulfane mixture was performed by ultra-performance liquid chromatography (UPLC) silver coordination ion spray mass spectrometry ((Ag^+^)CIS-MS) (Figure 2) as well as by ^19^F NMR and direct analysis in real time–mass spectrometry (DART-MS) techniques, as described more fully in the experimental section and elsewhere [55]. Reversed phase HPLC analysis of the mixture indicated the presence of higher polysulfides containing up to 26 sulfur atoms.

All new compounds were fully characterized by spectroscopic methods, either as pure compounds, or in the case of **18** mixed with **12**, **19** mixed with **20**, and **21**, as mixtures. While synthesis of fluorine-substituted analogs of garlic-derived organosulfur compounds in Scheme 1 is straightforward, their accessibility is limited by the high cost of starting material **9**. While **12** and **18** are chiral due to the sulfinyl group, thiosulfinates, such as **12,** are stereochemically labile [2,3], so no effort was made to synthesize enantiomerically pure material.

## 3. Materials and Methods

### 3.1. General Procedures

NMR spectra were recorded in CDCl_3_, unless otherwise indicated, on a 400 Ultrashield, spectrometer (Bruker, Billerica, MA, USA) operating at 400 MHz for proton, 100 MHz for carbon, and a Bruker 600 Avance III HD with QCI Cryoprobe operating at 600 MHz for proton, 125 MHz for carbon and 376.5 MHz for ^19^F. The chemical shifts (**δ**) are indicated in ppm downfield from tetramethylsilane, with the internal standard being the residual CHCl_3_ (δ 7.27 ppm for proton and δ 77.23 for carbon spectra). ^19^F-NMR chemical shifts were relative to CFCl_3_ at δ 0.00 ppm. Infrared spectra of the neat compounds were recorded on a UATR 2 FTIR (Perkin Elmer, Boston, MA USA). GC-MS were obtained using the Hewlett Packard 6890 GC and a 5972A selective mass detector (Agilent Technologies, Inc., Santa Clara, CA, USA). A DART-AccuTOF (JEOL USA, Inc., Peabody, MA, USA) time-of-flight mass spectrometer operating in positive or negative ion mode was employed with a polyethylene glycol spectrum as reference standard for exact mass measurements (PEG average molecular weight: 600). The atmospheric pressure interface was typically operated at the following potentials: orifice 1 = 15 V; orifice 2 = 5 V; ring lens = 3 V. The RF ion guide voltage was generally set to 800 V to allow detection of ions greater than *m*/*z* 80. The DART ion source was operated with helium gas at 200 °C, and a grid voltage = 530. 3-Chloro-2-fluoroprop-1-ene (**9**) was obtained from Synquest Laboratories (Alachua, FL, USA). Selected ^1^H-, ^13^C- and ^19^F-NMR and DART-MS data for new compounds are available in the Supporting Materials.

### 3.2. Synthesis of Fluorinated Garlic Organosulfur Compounds

*S-(2-Fluoro-2-propenyl)ethanethioate* (**10**). Caution: 3-chloro-2-fluoroprop-1-ene is toxic (LD_50_ for skin penetration, 200 mg/kg) and, therefore, should be handled with great care [45,57,58]. Potassium thioacetate (2.50 g, 22 mmol) was added to a solution of 3-chloro-2-fluoroprop-1-ene (**9**, 1.89 g, 20 mmol) in THF (50 mL). The mixture was stirred overnight to give an orange-yellow solution with white precipitate. The solution was passed through a silica-gel pad and then concentrated in vacuo to give 1.60 g (60%, 11.9 mmol) of the title compound **10** as a red liquid, which is pure enough for further use; ^1^H NMR (400 MHz, CDCl_3_) δ 4.64 (dd, ^3^J_HF_ = 15.6, ^2^J_HH_ = 3.1 Hz, CF=CH(Z), 1H), 4.54 (dd, ^3^J_HF_ = 43.9 Hz, ^2^J_HH_ = 3.1 Hz, CF=CH(E), 1H), 3.66 (d, ^3^J_HF_ = 17.2 Hz, C**H**_2_, 2H), 2.37 (s, C**H**_3_, 3H); ^13^C NMR (100 MHz, CDCl_3_) δ 194.2 (s), 161.6 (d, ^1^J_CF_ = 256 Hz), 93.1 (d, ^2^J_CF_ = 19.1 Hz), 30.7 (s), 30.0 (d, ^2^J_CF_ = 31.3 Hz); ^19^F NMR (376.5 MHz, CDCl_3_) δ −98.23 (m). IR: ν_max_ 2981, 1697, 1673, 1275, 1132, 1105, 944, 857, 846, 624 cm^−1^; HRMS (ESI) exact mass calculated for [M + H]^+^ (C_5_H_8_OFS) requires *m*/*z* 135.0279, found *m*/*z* 135.0290.

*1,2-Bis(2-fluoro-2-propenyl)disulfane* (**11**). Sodium chips (0.12 g, 5.0 mmol) were added to anhydrous methanol (25 mL) at 0 °C. The mixture was stirred until the sodium completely disappeared. Compound **10** (0.67 g, 5.0 mmol) was added to the above solution at 0 °C. Iodine (0.63 g, 2.5 mmol) was added to the solution after 2 h. The mixture was stirred for 30 min and then concentrated and purified by silica gel column chromatography (hexanes) to give the title compound as a light yellow liquid (0.24 g, 53%); ^1^H NMR (400 MHz, CDCl_3_) δ 4.72 (dd, *J* = 15.4, 3.0 Hz, 2H), 4.53 (dd, *J* = 47.5, 3.0 Hz, 2H), 3.41 (d, *J* = 18.9 Hz, 4H); ^13^C NMR (100 MHz, CDCl_3_) δ 161.1 (d, *J*_CF_ = 256 Hz), 94.5 (d, *J*_CF_ = 19.2 Hz), 40.4 (d, *J*_CF_ = 30.1 Hz); ^19^F NMR (376.5 MHz, CDCl_3_) δ −100.37 (m); IR: ν_max_ 2919, 1668, 1271, 1133, 946, 891, 855, 841, 732 cm^−1^; HRMS (ESI): calcd for C_6_H_9_F_2_S_2_ [M + H^+^] = 183.0114; found = 183.0121.

*Difluoroallicin (***12***; S-(2-Fluoroallyl) 2-fluoroprop-2-ene-1-sulfinothioate)*. A 32% peracetic acid/acetic acid solution (0.58 g, 2.6 mmol, 1.04 equiv.) was added dropwise to a solution of **11** (0.46 g, 2.5 mmol) in chloroform (50 mL) at 0 °C. The reaction mixture was vigorously stirred at 0 °C for 45 min as anhydrous Na_2_CO_3_ (4.0 g, 37.7 mmol, 15.1 equiv.) was added in small portions. The mixture was stirred for an additional 1 h at 0 °C and then filtered through a pad of Celite and anhydrous MgSO_4_. The filtrate was concentrated in vacuo at 0 °C to yield the crude, water-soluble title compound **12**, which was purified by preparative TLC (4:1 pentane:diethyl ether) to give pure **12** as yellow liquid with an onion-like odor (0.21 g, 1.06 mmol, 42%); ^1^H-NMR (400 MHz, CDCl_3_) **δ 4.97 (dd, *J* = 15, 3 Hz,** S(O)–CH_2_–CF=CH(Z), 1H), 4.78 (**dd, *J* = 14, 3 Hz,** S–CH_2_–CF=CH(Z), 1H), **4.70 (dd, *J* = 48, 3 Hz,** S(O)–CH_2_–CF=CH(E), 1H), **4.63 (dd, *J* = 47, 3 Hz,** S–CH_2_–CF=CH(E), 1H), 4.00–3.75 (m, 4H)); ^13^C-NMR (100 MHz, CDCl_3_) **δ** 32.5 (d, ^2^*J*_CF_ = 31.0 Hz) 58.8 (d, ^2^*J*_CF_ = 29.0 Hz), 94.1 (d, ^2^*J*_CF_ = 16.9 Hz) 98.3 (d, ^2^*J*_CF_ = 17.9 Hz), 155.4 (d, ^1^*J*_CF_ = 259 Hz), 160.5 (d, ^1^*J*_CF_ = 161 Hz); ^19^F-NMR (376.5 MHz, CDCl_3_) **δ** −99.65 to −99.44 (m, 1F), −94.90 to −94.62 (m, 1F); IR: ν_max_ 2917, 1669, 1407, 1272, 1081 (S=O), 945 cm^−1^; HRMS (ESI) exact mass calculated for [M + H]^+^ (C_6_H_9_OF_2_S_2_) requires *m*/*z* 199.0063, found *m*/*z* 199.0080. Storage at −40 °C or lower is recommended to avoid decomposition. Vacuum distillation was not attempted due to the presumed thermal sensitivity of **12**.

*S-2-Fluoro-2-propenyl-l-cysteine (***13***; 2-Amino-3-((2-fluoroallyl)thio)propanoic acid)*. 3-Chloro-2-fluoroprop-1-ene (**9**) (0.94 g, 10 mmol) was added to an ice-cold solution of l-cysteine (1.21 g, 10 mmol) in ammonium hydroxide (40 mL) with vigorous stirring. The mixture was stirred at 0 °C for 60 min and filtered; the filtrate was concentrated in vacuo (<40 °C) to a small volume and filtered. The solid was washed repeatedly with ethanol, dried in vacuo, and recrystallized from 2:3 water/ ethanol to yield **13** as white needles (1.31 g, 7.31 mmol, 73% yield), mp 220–223 °C; ^1^H-NMR (400 MHz, D_2_O): **δ** 4.48 (dd, J = 3.2, 12 Hz, 1H), 4.58 (dd, J = 3.2, 50 Hz, 1H), 3.90 (dd, J = 4, 8 Hz, 1H), 3.32 (d, 2H, *J* = 16 Hz), 3.13–2.99 (m, 2H); ^19^F-NMR (D_2_O): **δ** −101.30 to −101.58 (m, 1F); ^13^C-NMR (100 MHz, D_2_O): **δ** 172.41, 161.73, 159.21, 53.27, 31.63–31.35 (d), 31.16. Single crystals were analyzed by X-ray crystallography as described in Section 3.3 below to provide the crystal structure of the l-enantiomer shown in Figure 1.

*Trifluoroajoene* (**18**) *[(E,Z)-1-(2-Fluoro-3-((2-fluoroallyl)sulfinyl)prop-1-en-1-yl)-2-(2-fluoroallyl)disulfane]*. A solution of **12** (200 mg; 1.01 mmol) was dissolved in 1,1,1,3,3,3-hexafluoro-2-propanol (0.50 mL) at room temperature. The mixture was immediately analyzed by DART-MS which showed the formation of trace amounts of trifluoroajoene **18**. The mixture was stirred at room temperature for 16 h whereupon analysis by DART-MS showed a 1:1 mixture of F-allicin and F-ajoene, with no F-dithiin present. The mixture was concentrated in vacuo to yield a faintly yellow liquid (180 mg). ^1^H-, ^13^C- and ^19^F-NMR spectroscopy indicated a 1:1 mixture of difluoroallicin **12** and trifluoroajoene **18**, with no difluorodithiins present, corresponding to a 59% yield of **18** based on **12** converted. A small sample was stirred for 32 h but no further conversion of **12** to **18** was observed. Attempts to purify **18** by silica gel chromatography or HPLC were unsuccessful; ^1^H-NMR (400 MHz, CDCl_3_) **18**: δ 5.91 (d, 2H, ^3^J_HF_ = 120.9), 5.90 (s, 1H), 4.4 (m, 2H), 3.8 (m, 6H); **12** as above; ^13^C-NMR (100 MHz, CDCl_3_) **18**: δ 59.75 (d, 1C, *J* = 27.70 Hz), 60.02 (d, 1C, *J* = 27.59 Hz), 68.82 (d, 1C, *J* = 33.33 Hz), 69.32 (s, 1C), 69.81 (d, 1C, *J* = 33.23 Hz), 109.44 (d, 1C, *J* = 36.08 Hz), 108.57 (d, 1C, *J* = 283.20 Hz), 110.86 (d, 1C, *J* = 247.17 Hz), 121.75 (d, 1C, *J* = 278.34 Hz); **12** as above; ^19^F-NMR (376.5 MHz, CDCl_3_) **18**: δ −78 (s, 1F), −128 (s,1F), −140 (s, 1F); **12** as above; HRMS (ESI) exact mass calculated for [M + H]^+^ (C_9_H_12_F_3_OS_3_) requires *m*/*z* 289.0002, found *m*/*z* 288.9992.

*5-Fluoro-3-(1-fluorovinyl)-3,4-dihydro-1,2-dithiin* (**16**). A solution of **12** (50.0 mg) in CHCl_3_ (1 mL) was placed in a glass tube which was sealed under vacuum and heated at 80 °C for 4.0 h. The reaction mixture was analyzed by GC-MS and the major dithiin isomer **16** was purified by flash chromatography (*n*-pentane); ^1^H-NMR (400 MHz, CDCl_3_) δ 3.61–2.84 (m, 2H), 3.93–3.99 (m, 1H), 4.44–4.57 (m, 1H), 4.39–4.94 (m, 1H), 6.09–6.13 (m, 1H). ^13^C-NMR (100 MHz, CDCl_3_) δ 30.3 (dd, *J* = 28.0, 5.0 Hz), 45.5 (dd, *J* = 30.0, 11.5 Hz), 93.3 (d, *J* = 19.5 Hz), 101.0 (d, *J* = 27.0 Hz) 157.0 (d, *J* = 395.0 Hz) 160.0 (d, *J* = 395.0 Hz); ^19^F-NMR (376.5 MHz CDCl_3_): δ −99.80 to −99.60 (m, 1F), −88.83 to −88.78 (m, 1F). IR: ν_max_ 2853, 1668, 1420, 1257, 1192, 1100, 928 cm^−1^; DART-HR-TOF-MS [M + H]^+^ C_6_H_7_F_2_S_2_ found *m*/*z* 180.9958, requires *m*/*z* 180.9957.

*2-Fluoroacrolein-ethyl acrylate Diels-Alder adducts: Ethyl 5-fluoro-3,4-dihydro-2H-thiopyran-2-carboxylate (***19**) *and ethyl 5-fluoro-3,4-dihydro-2H-thiopyran-3-carboxylate* (**20**). A mixture of difluoroallicin **12** (98.0 mg, 0.5 mmol) and ethyl acrylate (250.0 mg, 2.5 mmol) in CHCl_3_ (2.5 mL) were placed in a glass tube. The tube was sealed under vacuum and heated at 100 °C for 6.0 h. The reaction mixture was analyzed by NMR and the crude material was purified by using flash chromatography (n-pentane/Et_2_O), affording 70:30 (**19**:**20**) mixture (56.0 mg, 60% yield). It was not possible to separate completely **19** (major) from **20** (minor) by flash chromatography. DART-HR-TOF-MS (M + H) C_8_H_12_FO_2_S found *m*/*z* 191.0538, requires *m*/*z* 191.0542. **19** (Major 70%): ^1^H-NMR (400 MHz, CDCl_3_) δ 5.67 (d, J = 17.7 Hz, 1H), 4.15–4.22 (m, 2H, overlap with **20**), 3.05–3.08 (m, 1H), 2.85–2.98 (m, 2H overlap with **20**), 2.49–2.63 (m, 2H, overlap with **20**), 1.26–1.30 (m, 3H, overlap with **20**); ^13^C-NMR (100 MHz, CDCl_3_: δ 172.0, 153.5 (d, J = 259.7 Hz), 97.3 (d, J = 27.5 Hz), 61.2, 40.7 (m, overlap with **20**), 27.2 (m, overlap with **20**), 26.4, 14.0 (overlap with **20**); ^19^F-NMR (376.5 MHz CDCl_3_): δ −96.26 to −96.21 (m). **20** (Minor 30%): ^1^H-NMR (400 MHz, CDCl_3_) δ 5.61 (d, J = 17.7 Hz, 1H), 4.15–4.22 (m, 2H, overlap with **19**), 3.35–3.43 (m, 1H), 2.85–2.98 (m, 2H), 2.49–2.63 (m, 2H, overlap with **19**), 1.26–1.30 (m, 3H, overlap with **19**); ^13^C-NMR (100 MHz, CDCl_3_) δ 170.4, 151.4 (d, J = 259.7 Hz), 95.7 (d, J = 27.5 Hz), 61.6, 40.6 (m, overlap with **19**), 27.2 (m, overlap with **19**), 23.4, 14.0 (overlap with **19**); ^19^F-NMR (376.5 MHz CDCl_3_): δ −96.09 to −96.04 (m).

*Bis(2-fluoroallyl) polysulfane mixture* (**21**). A 10 mL round-bottomed flask containing sublimed sulfur (S_8_, 0.256 g, 1.0 mmol) was placed in an oil bath pre-heated to 120 °C. When the sulfur had completely melted to a clear, straw-colored liquid, disulfide **11** (0.182 g, 1.0 mmol) was added all at once to the magnetically stirred liquid. After 30 min, the initial two-layer liquid mixture became a clear, homogeneous solution. Samples were withdrawn from the reaction mixture for analysis at various time points, e.g., 30 min, 1 h, 2 h, 4 h and 5 h. The withdrawn samples were dissolved in CDCl_3_ permitting both NMR and reversed phase (RP) HPLC analysis to be performed on the same sample. For the 2 h sample, disulfide **11** eluted at 2.65 min, S_8_ eluted between the heptasulfide and octasulfide at 22.2 min, and the highest polysulfane seen, eluting at 159 min, had a chain of 26 sulfur atoms. By UPLC-(Ag^+^)-CIS-MS, chains containing up to 15 sulfur atoms could be characterized as their ^107^Ag and ^109^Ag adducts (see Supporting Information and discussion elsewhere [53]). ^1^H-NMR spectroscopic analysis after 30 min shows the CH_2_ proton signals as doublets centered at δ 3.39 [area 0.35, S2], 3.52 [area 0.08, S3], 3.61 [area 0.08, S4], 3.63 [area 0.16, S5], 3.64 [area 0.50, ≥S6]; after 5 h ^1^H-NMR spectroscopic analysis shows 3.39 [area 0.06 S2], 3.52 [area 0.08 S3], and 3.64 [area 0.95, ≥S6]. After 1.25 h, ^19^F-NMR (376.5 MHz CDCl_3_) showed a series of multiplets centered at δ −100.3 (**11**; C_6_H_8_F_2_S_2_) and −100.0 (C_6_H_8_F_2_S_3_) down to −99.5 ppm (C_6_H_8_F_2_S_>5_). DART was used to obtain HRMS (ESI) exact mass data on C_6_H_9_F_2_S_n_ components in a mixture examined after 1.25 h of heating. The lighter components gave better exact mass agreement compared to the heavier components: calculated for [M + H]^+^ (**11**; C_6_H_9_F_2_S_2_) requires *m*/*z* 183.0114, found *m*/*z* 183.0117; calculated for [M + H]^+^ (C_6_H_9_F_2_S_3_) requires *m*/*z* 214.9835, found *m*/*z* 214.9835; calculated for [M + H]^+^ (C_6_H_9_F_2_S_4_) requires *m*/*z* 246.9555, found *m*/*z* 246.9563; calculated for [M + H]^+^ (C_6_H_9_F_2_S_5_) requires *m*/*z* 278.9276, found *m*/*z* 278.9242; calculated for [M + H]^+^ (C_6_H_9_F_2_S_6_) requires *m*/*z* 310.8997, found *m*/*z* 310.8985; calculated for [M + H]^+^ (C_6_H_9_F_2_S_7_) requires *m*/*z* 342.8717, found *m*/*z* 342.8808; calculated for [M + H]^+^ (C_6_H_9_F_2_S_8_) requires *m*/*z* 374.8438, found *m*/*z* 374.9639; calculated for [M + H]^+^ (C_6_H_9_F_2_S_9_) requires *m*/*z* 406.8159, found *m*/*z* 406.9403; calculated for [M + H]^+^ (C_6_H_9_F_2_S_10_) requires *m*/*z* 438.7880, found *m*/*z* 438.9084. UPLC-(Ag^+^)-CIS-MS analysis of (**21**) is listed in Table S2.

*(+)-S-2-Fluoro-2-propenyl-l-cysteine S-oxide* (**22**). With vigorous stirring, **13** (1.18 g, 6.59 mmol) was dissolved in 12 mL of water. Hydrogen peroxide (30%, 2.56 g) was added to the solution which was then stirred for 45 min at RT. The water was removed under vacuum, and the title compound **22** (1.04 g, 82%, 5.33 mmol) was obtained as a white powder, mp 168–171 °C; ^1^H-NMR (D_2_O) δ 5.03 (dd, 2H, *J* = 3 Hz), 4.86–4.82 (m, 2H) 4.08–3.75 (m, 2H), 3.50–3.26 (m, 2H); ^19^F-NMR (D_2_O) δ −94.84 to −95.12 (m, 1F), −95.18 to −95.47 (m, 1F).

### 3.3. X-Ray Crystallographic Structural Determination for **13**

The single crystal diffraction data for 13 was measured on a SMART APEX CCD X-ray diffractometer (Bruker, Madison, WI, USA) equipped with a graphite monochromated Mo Kα radiation source (*λ* = 0.71073 Å) at T = 100(2) K. Data reduction and integration were performed with the Bruker software package SAINT (Version 8.38A). Data were corrected for absorption effects using the empirical methods as implemented in SADABS. Both SAINT and SADABS are part of Bruker APEX3 software package (Version 2016.9-0): Bruker AXS, 2016. The structure was solved by SHELXT (Version 2014/5) [59] and refined by full-matrix least-squares procedures using the Bruker SHELXTL (Version 2016/6) XL refinement program version 2016/6 software package [59]. All non-hydrogen atoms (including those in disordered parts) were refined anisotropically. The H-atoms were also included at calculated positions and refined as riders, with *U*_iso_(H) = 1.2 *U*_eq_(C) and *U*_iso_(H) = 1.5 *U*_eq_(N) for ammonia groups. Crystallographic data, details of the data collection and structure refinement for **13** are listed in Table S1.

## 4. Conclusions

We describe here the synthesis and characterization of a series of new fluorinated derivatives of key, biologically active garlic-derived organosulfur compounds, including difluoroallicin (**12**). Upon heating, **12** decomposes to 2-fluorothioacrolein (**15**), a previously unknown highly reactive fluorine-substituted heterodiene, trapped with ethyl acrylate to afford a mixture of isomeric 5- and 6-substituted 3-fluoro-5,6-dihydro-4*H*-thiopyrans. Our findings suggest a useful new synthetic route to fluorine-substituted thiopyrans, of potential pharmaceutical interest. Several of the other fluorinated organosulfur compounds prepared are anticipated to show interesting biological activity. Further studies on these new compounds and preparation of more heavily fluorinated homologues seem warranted.

### X-Ray Crystallographic Data

CCDC1577315 contains the supplementary crystallographic data, which can be obtained free of charge from Cambridge Crystallographic Data Centre, www.ccdc.cam.ac.uk/data_request/cif (accessed on 20 June 2025).

## Figures and Tables

**Table 1:** Selected bond distances (Å) and angles (deg.) in **13**. Atom labeling as in Figure 1.

Bond Lengths					
S(1)-C(10)	1.806(3)	C(10)-C(11)	1.472(5)	F(1)-C(5)	1.360(4)
S(1)-C(9)	1.812(3)	C(9)-C(8)	1.512(5)	C(1)-C(2)	1.524(4)
O(5)-C(7)	1.242(4)	C(11)-C(12)	1.287(5)	C(5)-C(6)	1.286(5)
O(4)-C(7)	1.238(4)	S(2)-C(4)	1.803(4)	C(5)-C(4)	1.477(5)
N(2)-C(8)	1.479(4)	S(2)-C(3)	1.815(3)	C(2)-N(1)	1.488(4)
F(2)-C(11)	1.362(4)	O(1)-C(1)	1.242(4)	C(2)-C(3)	1.510(4)
C(7)-C(8)	1.525(4)	O(2)-C(1)	1.247(4)		
Angles					
C(4)-S(2)-C(3)	100.4(2)	C(6)-C(5)-F(1)	119.4(3)	N(1)-C(2)-C(1)	109.6(3)
O(1)-C(1)-O(2)	125.8(3)	C(6)-C(5)-C(4)	129.0(4)	C(3)-C(2)-C(1)	112.4(3)
O(1)-C(1)-C(2)	118.5(3)	F(1)-C(5)-C(4)	111.5(3)	C(2)-C(3)-S(2)	113.4(2)
O(2)-C(1)-C(2)	115.7(3)	N(1)-C(2)-C(3)	110.8(3)	C(5)-C(4)-S(2)	113.4(3)

## Data Availability

Data are contained within the article and Supplementary Materials.

## Supplementary Materials

The following supporting information can be downloaded at https://www.mdpi.com/article/10.3390/molecules30132841/s1: Table S1: Documentation for the X-ray structure; Table S2: UPLC-(Ag^+^)-CIS-MS analysis of bis(2-fluoro-2-propenyl) polysulfanes (**21**). Selected ^1^H-, ^13^C- and ^19^F-NMR and DART-MS data for new compounds are available online.
