# Supplementary material for: Fluorinated Analogs of Organosulfur Compounds from Garlic (Allium sativum): Synthesis and Chemistry
Source: Molecules. 2025 Jul 2;30(13):2841. doi: 10.3390/molecules30132841 (PMC12251486; doi:10.3390/molecules30132841)

Supplementary Materials

Fluorinated Analogs of Organosulfur Compounds from Garlic (*Allium sativum*): Synthesis and Chemistry

Eric Block, Benjamin Bechand, Sivaji Gundala, Abith Vattekkatte and Kai Wang

TABLE OF CONTENTS

Table S1. Crystal data and structure refinement for **13**..2

Ultra-performance liquid chromatography silver coordination ion spray mass spectrometry of bis(2-fluoroallyl) polysulfane mixture (**21**)..3

Table S2. UPLC-(Ag^+)-CIS-MS Analysis of Bis(2-fluoro-2-propenyl) Polysulfanes (**21**)........4

^{1}H NMR Spectra..7
S-(2-Fluoro-2-propenyl)ethanethioate (**10**)..7
1,2-Bis(2-fluoro-2-propenyl)disulfane (**11**)..8
1,2-Bis(2-fluoroallyl)disulfane (**11**), enlargement...9
Difluoroallicin (**12**; *S*-(2-Fluoroallyl) 2-fluoroprop-2-ene-sulfinothioate)............................10
Difluoroallicin (**12**; *S*-(2-Fluoroallyl) 2-fluoroprop-2-ene-sulfinothioate) enlargement... 11
1,2-Bis(2-fluoro-2-propenyl)polysulfane (**21**)..12
S-2-Fluoro-2-propenyl-L-cysteine (**13**)...13

^{13}C NMR Spectra...14
Difluoroallicin (**12**; *S*-(2-Fluoro-2-propene) 2-fluoroprop-2-ene-sulfinothioate)...................14
S-(2-Fluoro-2-propenyl) ethanethioate (**10**)..15
1,2-Bis(2-fluoro-2-propenyl)disulfane (**11**)..16
S-2-Fluoro2-propenyl-L-cysteine (**13**)...17

^{19}F NMR Spectra..18
Difluoroallicin (**12**; *S*-(2-Fluoroallyl) 2-fluoroprop-2-ene-sulfinothioate)..........................18
S-(2-Fluoro-2-propenyl) ethanethioate (**10**)..19
1,2-Bis(2-fluoro-2-propenyl) disulfane (**11**)...20
Mixture of difluoroallicin, **12**, and trifluoroajoene, **18**...21
S-2-Fluoro-2-propenyl-L-cysteine (**13**)(fluorodeoxyalliin)..22
(+)-*S*-2-Fluoro-2-propenyl-L-cysteine *S*-oxide (**22**)..23

DART Mass Spectra...24

Table S1. Crystal data and structure refinement for **13**.

Compound	str949
Empirical formula	$C_6H_{10}FNO_2S$
Formula weight	179.21
Temperature (K)	100(2)
Wavelength (Å)	0.71073
Crystal system	Monoclinic
Space group	$P2_1$
a (Å)	9.343(3)
b (Å)	5.2055(17)
c (Å)	16.632(6)
α (°)	90.00
β (°)	92.240(5)
γ (°)	90.00
V (Å^3)	808.3(5)
Z	4
ρ_{calcd} (g·cm^{-3})	1.473
μ (mm^{-1})	0.368
F(000)	376
Crystal size (mm)	0.12×0.08×0.02
ϑ range for data collection (°)	2.18-27.04
Reflections collected	6653
Independent reflections	3453 [R_{int} = 0.0370]
Transmission factors (min/max)	0.9642/1
Data/restraints/params.	3453/1/199
Flack x parameter	-0.01(8)
$R1$,[a] $wR2$[b] ($I > 2\sigma(I)$)	0.0497, 0.1012
$R1$,[a] $wR2$[b] (all data)	0.0701, 0.1111
Quality-of-fit[c]	0.982

[a]$R1 = \Sigma||F_o|-|F_c||/\Sigma|F_o|$. [b]$wR2 = [\Sigma[w(F_o^2-F_c^2)^2]/\Sigma[w(F_o^2)^2]]$.
[c]Quality-of-fit = $[\Sigma[w(F_o^2-F_c^2)^2]/(N_{obs}-N_{params})]^{½}$, based on all data.

Ultra-performance liquid chromatography silver coordination ion spray mass spectrometry of bis(2-fluoroallyl) polysulfane mixture (**21**)

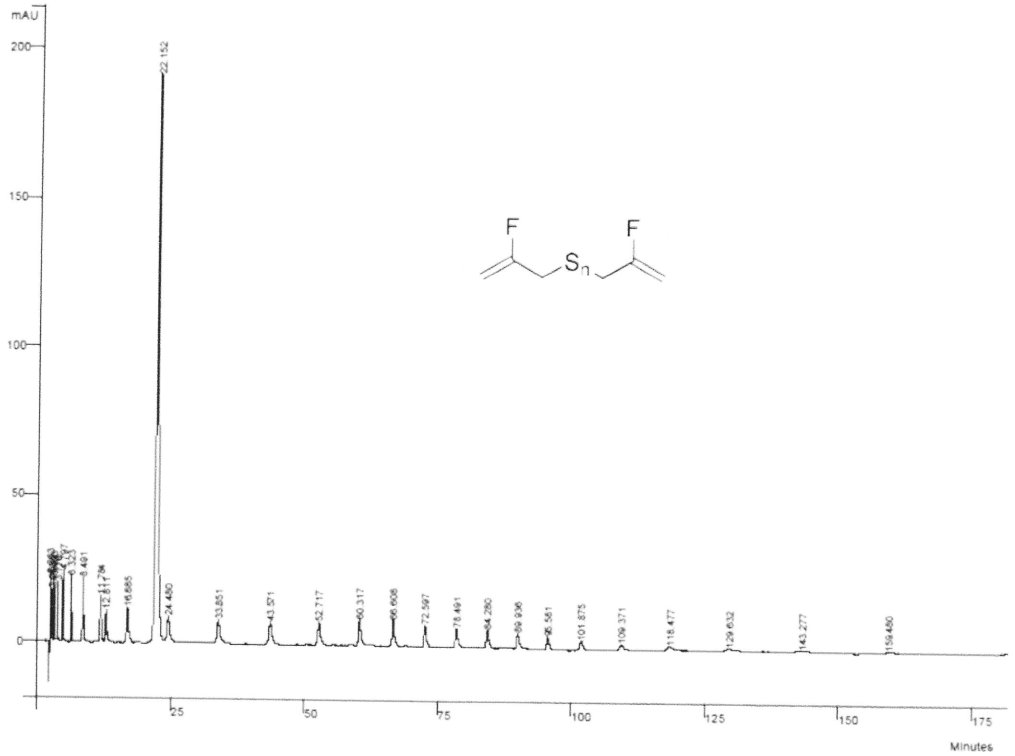

Table S2. UPLC-(Ag^+)-CIS-MS Analysis of Bis(2-fluoro-2-propenyl) Polysulfanes (21)

Calcd smallest parent mass (exact): 352.853 Da *Found: 352.802 Da* **S4**
Mass of tallest peak: 355 Da [3.53 min]
Formula: $\mathbf{C_6H_8F_2S_4Ag}$

m/z	%, calcd.	*m/z* measured	%, measured
353	89.7	352.8	95.3
355	100	354.79	100
357	16.7	356.87	14.7

Calcd smallest parent mass (exact): 384.825 Da *Found: 384.820 Da* **S5**
Mass of tallest peak: 387 Da [4.55 min]
Formula: $\mathbf{C_6H_8F_2S_5Ag}$

m/z	%, calcd.	*m/z* measured	%, measured
385	86.2	384.82	81.4
387	100	386.8	100
389	20.5	388.79	23.2
391	1.9	390.83	2.3

Calcd smallest parent mass (exact): 416.797 Da *Found: 416.777 Da* **S6**
Mass of tallest peak: 418 Da [5.64 min]
Formula: $\mathbf{C_6H_8F_2S_6Ag}$

m/z	%, calcd.	*m/z* measured	%, measured
416	82.9	416.78	79.8
418	100	418.77	100
420	24.1	420.69	25.2
422	2.8	422.75	2.9
424	0.2	424.64	0.35

Calcd smallest parent mass (exact): 448.769 Da *Found: 448.712 Da* **S7**
Mass of tallest peak: 450 Da [6.50 min]
Formula: $\mathbf{C_6H_8F_2S_7Ag}$

m/z	%, calcd.	*m/z* measured	%, measured
448	79.8	448.71	73.8
450	100	450.71	100
452	27.7	452.65	29.0
454	3.8	454.71	4.2

Calcd smallest parent mass (exact): 480.741 Da *Found: 480.659* **S8**
Mass of tallest peak: 482 Da [7.56 min]
Formula: $\mathbf{C_6H_8F_2S_8Ag}$

m/z	%, calcd.	*m/z* measured	%, measured
480	77.0	480.66	73.6
482	100	482.64	100

484	31.2	484.58	34.9
486	4.9	486.66	5.1

Calcd smallest parent mass (exact): 512.7131 Da *Found: 512.607 Da* **S9**
Mass of tallest peak: 514 Da [8.47 min]
Formula: $\mathbf{C_6H_8F_2S_9Ag}$

m/z	%, calcd.	*m/z* measured	%, measured
512	74.4	512.6	73.4
514	100	514.6	100
516	34.6	516.5	38.4
518	6.2	518.5	7.4

Calcd smallest parent mass (exact): 362.682 Da *Found: 362.689 Da* **sulfur**
Mass of tallest peak: 364 Da [8.99 min]
Formula: $\mathbf{S_8Ag}$

m/z	%, calcd.	*m/z* measured	%, measured
362	77.4	362.69	79.3
364	100	364.68	100
366	30.7	366.68	34.9
368	4.7	368.7	4.6

Calcd smallest parent mass (exact): 544.685 Da *Found: 544,547 Da* **S10**
Mass of tallest peak: 546. Da [9.24 min]
Formula: $\mathbf{C_6H_8F_2S_{10}Ag}$

m/z	%, calcd.	*m/z* measured	%, measured
544	71.9	544.6	75.4
546	100	546.6	100
548	38.0	548.5	43.2
550	7.5	550.6	6.3

Calcd smallest parent mass (exact): 576.657 Da *Found: 576.523 Da* **S11**
Mass of tallest peak: 578. Da [9.99 min]
Formula: $\mathbf{C_6H_8F_2S_{11}Ag}$

m/z	%, calcd.	*m/z* measured	%, measured
576	69.6	576.5	67.7
578	100	578.5	100
580	41.3	580.4	44.7
582	9.0	582.5	7.0

Calcd smallest parent mass (exact): 608.629 Da *Found: 608.536 Da* **S12**
Mass of tallest peak: 610. Da [10.78 min]
Formula: $\mathbf{C_6H_8F_2S_{12}Ag}$

m/z	%, calcd.	*m/z* measured	%, measured
608	67.4	608.6	60.5
610	100	610.5	100

612	44.5	612.5	45.2

Calcd smallest parent mass (exact): 640.601 Da *Found: 640.365 Da* **S13**
Mass of tallest peak: 642 Da [11.63 min]
Formula: $\mathbf{C_6H_8F_2S_{13}Ag}$

m/z	%, calcd.	*m/z* measured	%, measured
640	65.330	640.4	62.0
642	100	642.4	100
644	47.7	644.4	48.8
646	12.3	646.3	9.3

Calcd smallest parent mass (exact): 672.574 Da *Found: 672.385 Da* **S14**
Mass of tallest peak: 674 Da [12.75 min]
Formula: $\mathbf{C_6H_8F_2S_{14}Ag}$

m/z	%, calcd.	*m/z* measured	%, measured
672	63.4	672.4	67.8
674	100	674.4	100
676	50.8	676.3	47.0

Calcd smallest parent mass (exact): 704.546 Da *Found: 704.310 Da* **S15**
Mass of tallest peak: 706 Da [14.48 min]
Formula: $\mathbf{C_6H_8F_2S_{15}Ag}$

m/z	%, calcd.	*m/z* measured	%, measured
704	61.6	704.3	51.2
706	100	706.3	100
708	53.9	708.3	60.2

^{1}H NMR Spectra

S-(2-Fluoro-2-propenyl)ethanethioate (**10**)

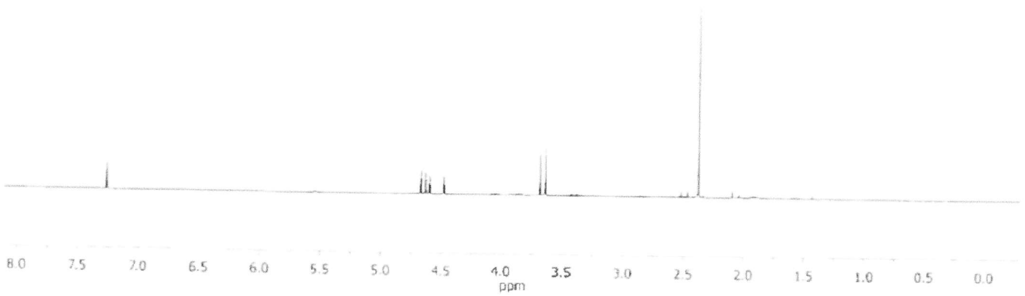

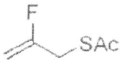

10

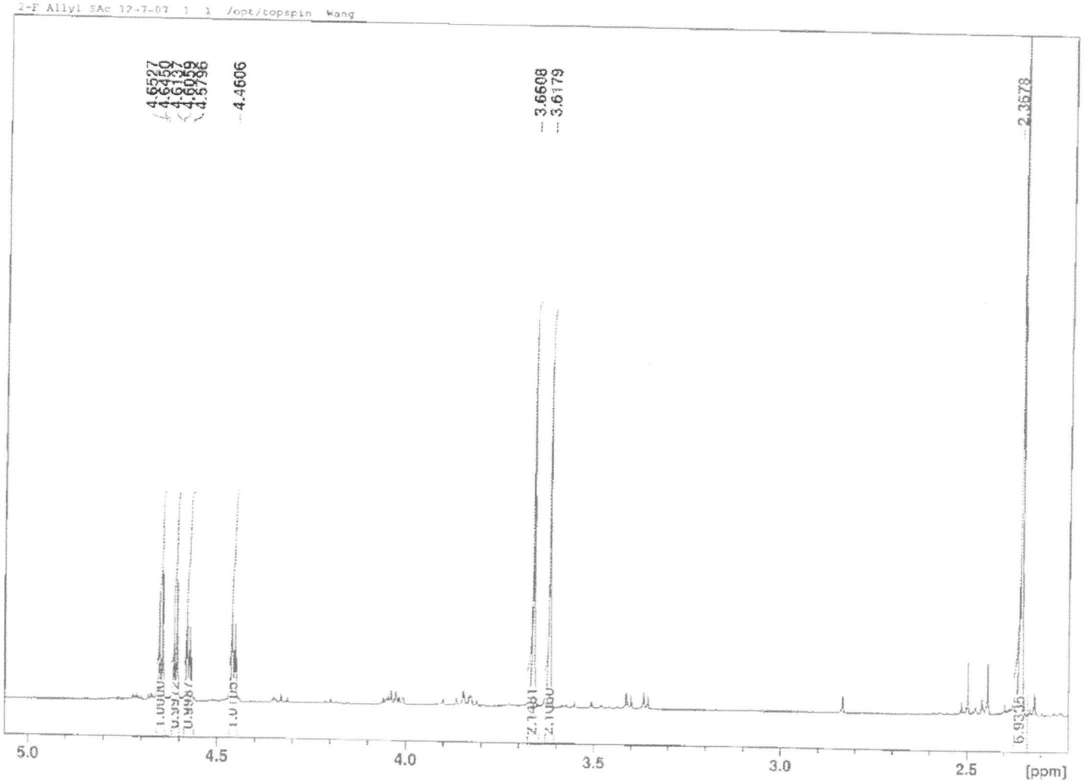

1,2- Bis(2-fluoro-2-propenyl)disulfide (**11**)

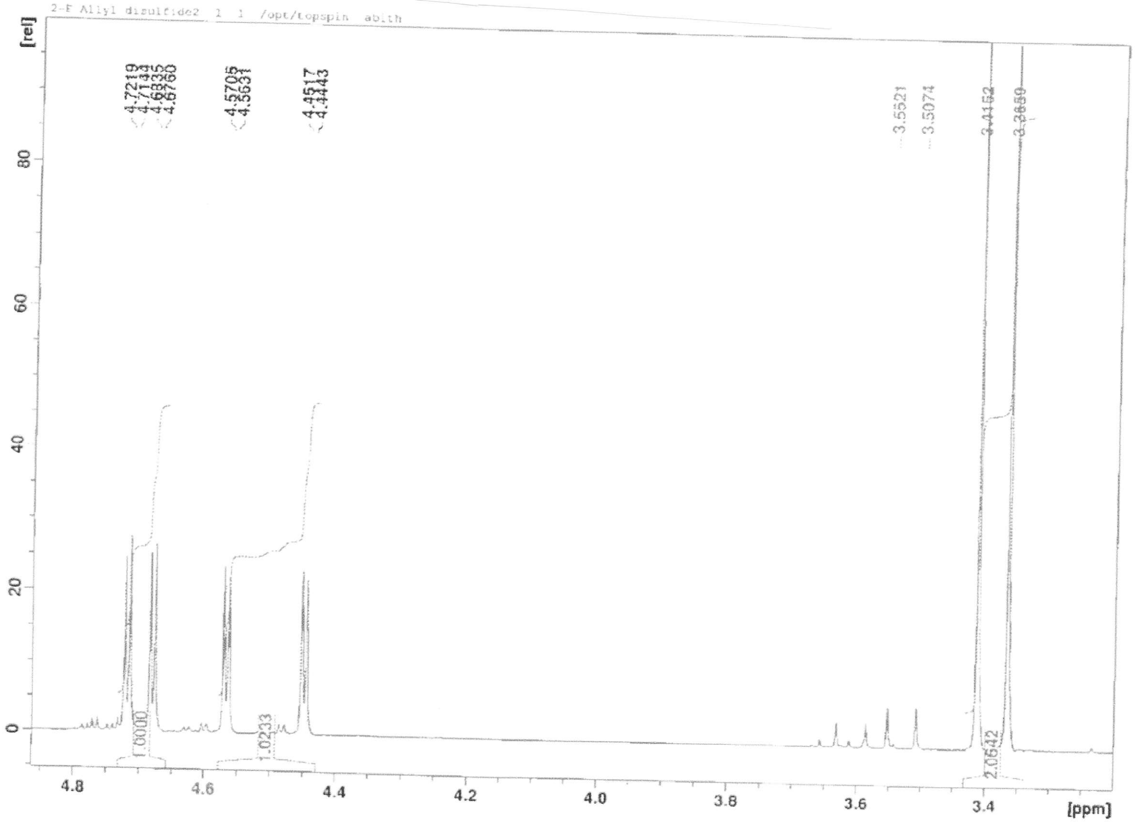

1,2- Bis(2-fluoro-2-propenyl)disulfide (**11**)

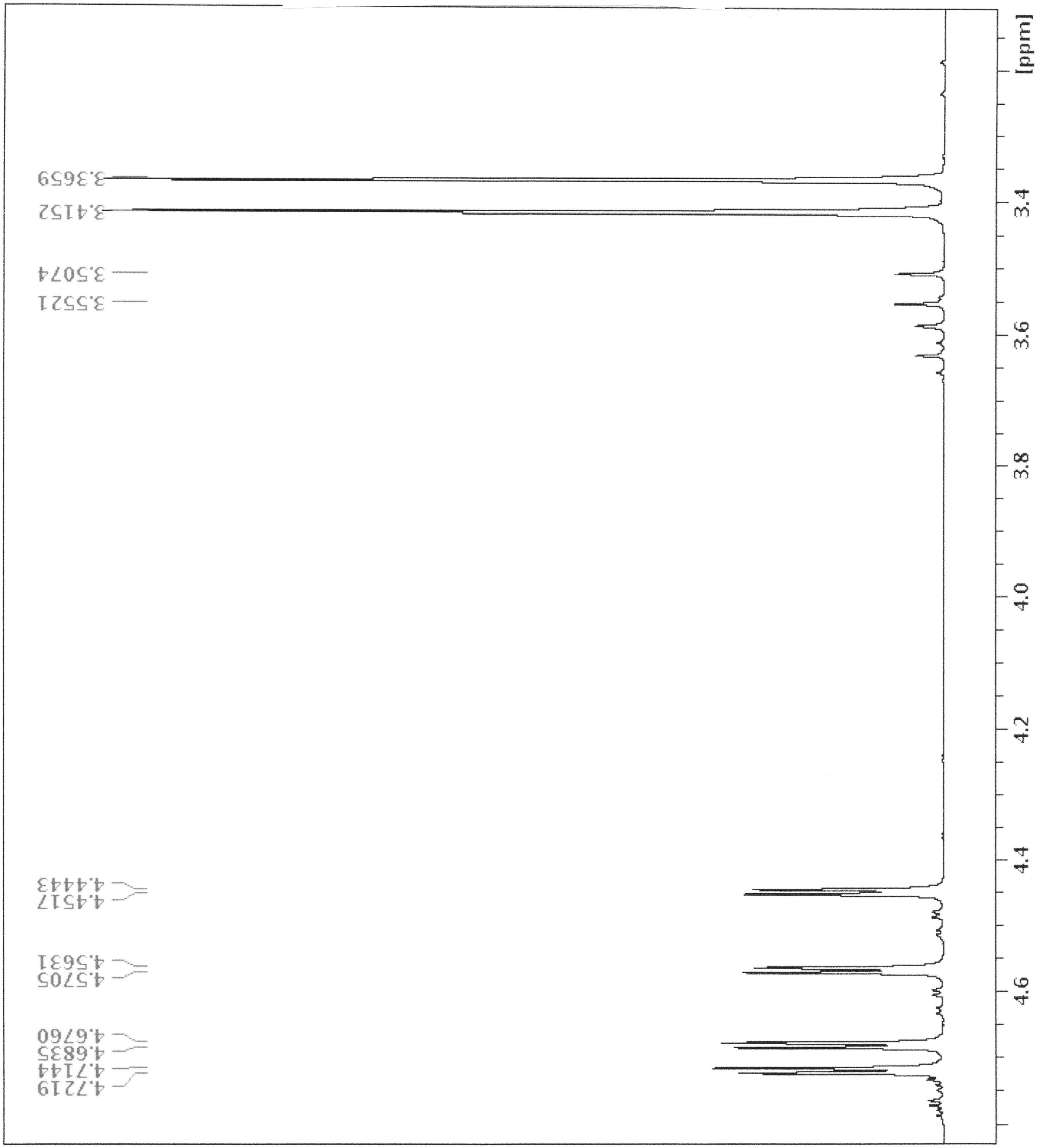

Difluoroallicin (**12**; *S*-(2-Fluoroallyl) 2-fluoroprop-2-ene-sulfinothioate)

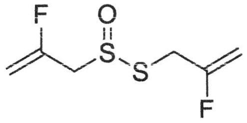

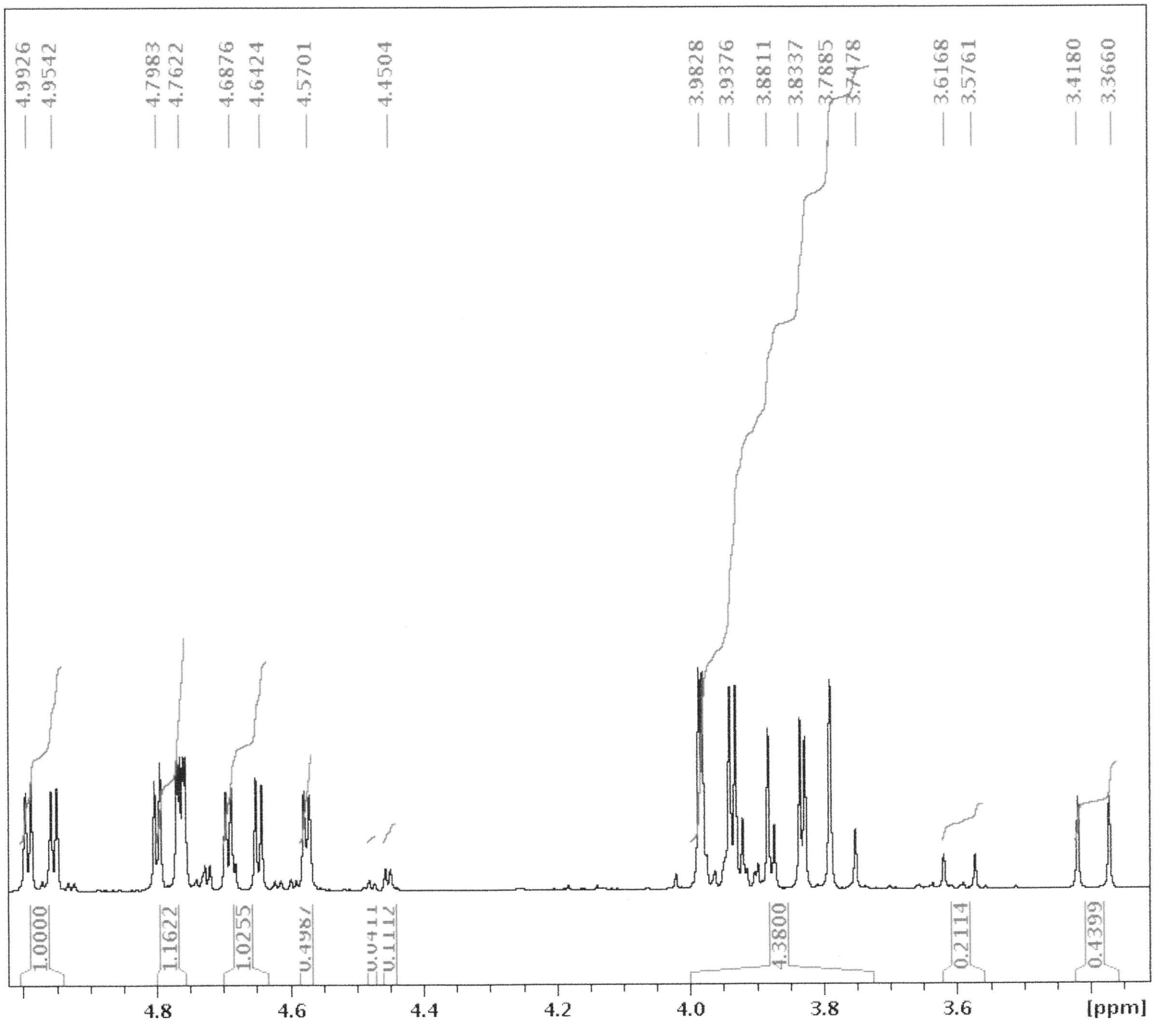

Difluoroallicin (**12**; *S*-(2-Fluoroallyl) 2-fluoroprop-2-ene-sulfinothioate)

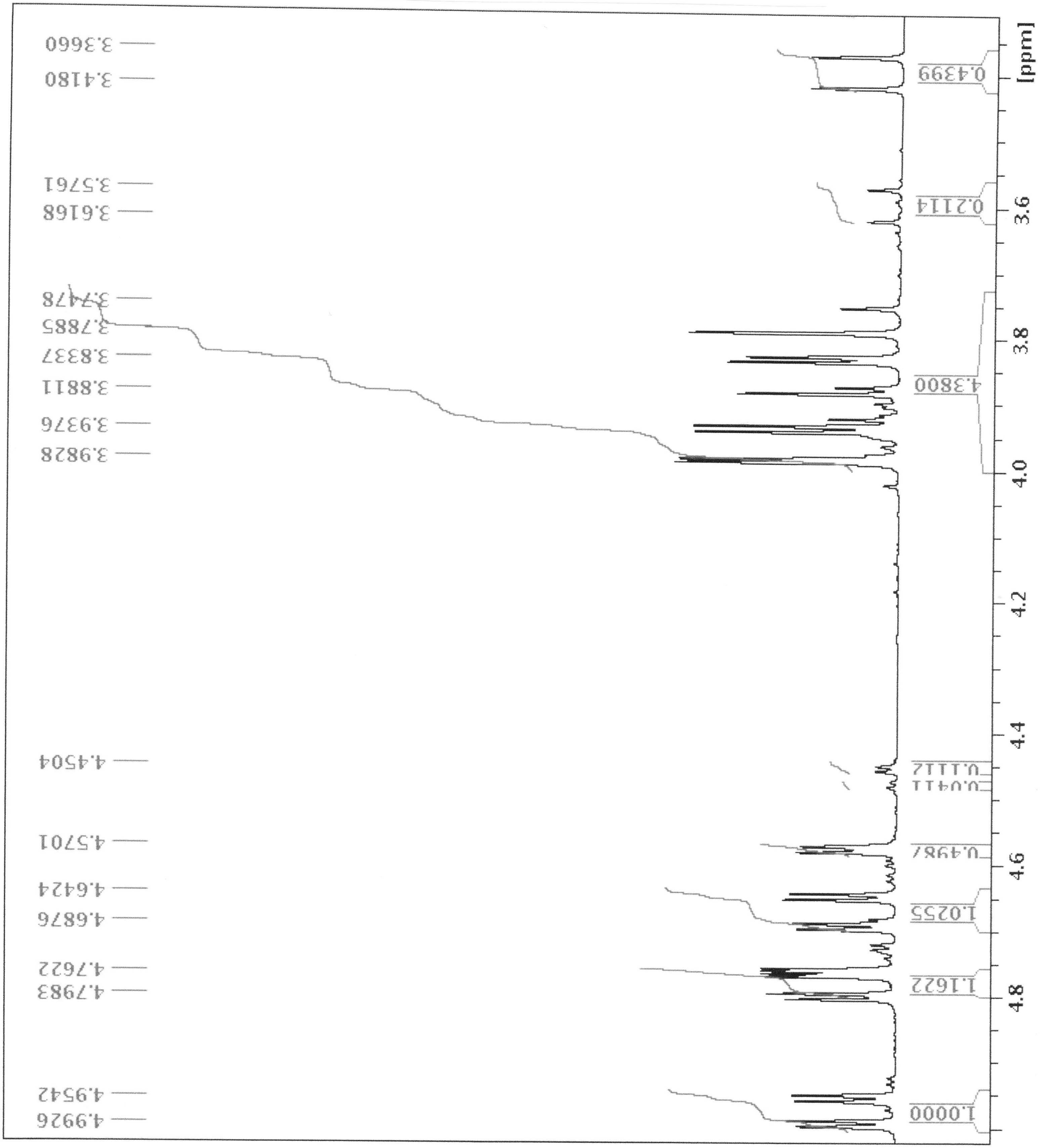

1,2-Bis(2-fluoro-2-propenyl)polysulfane (**21**)

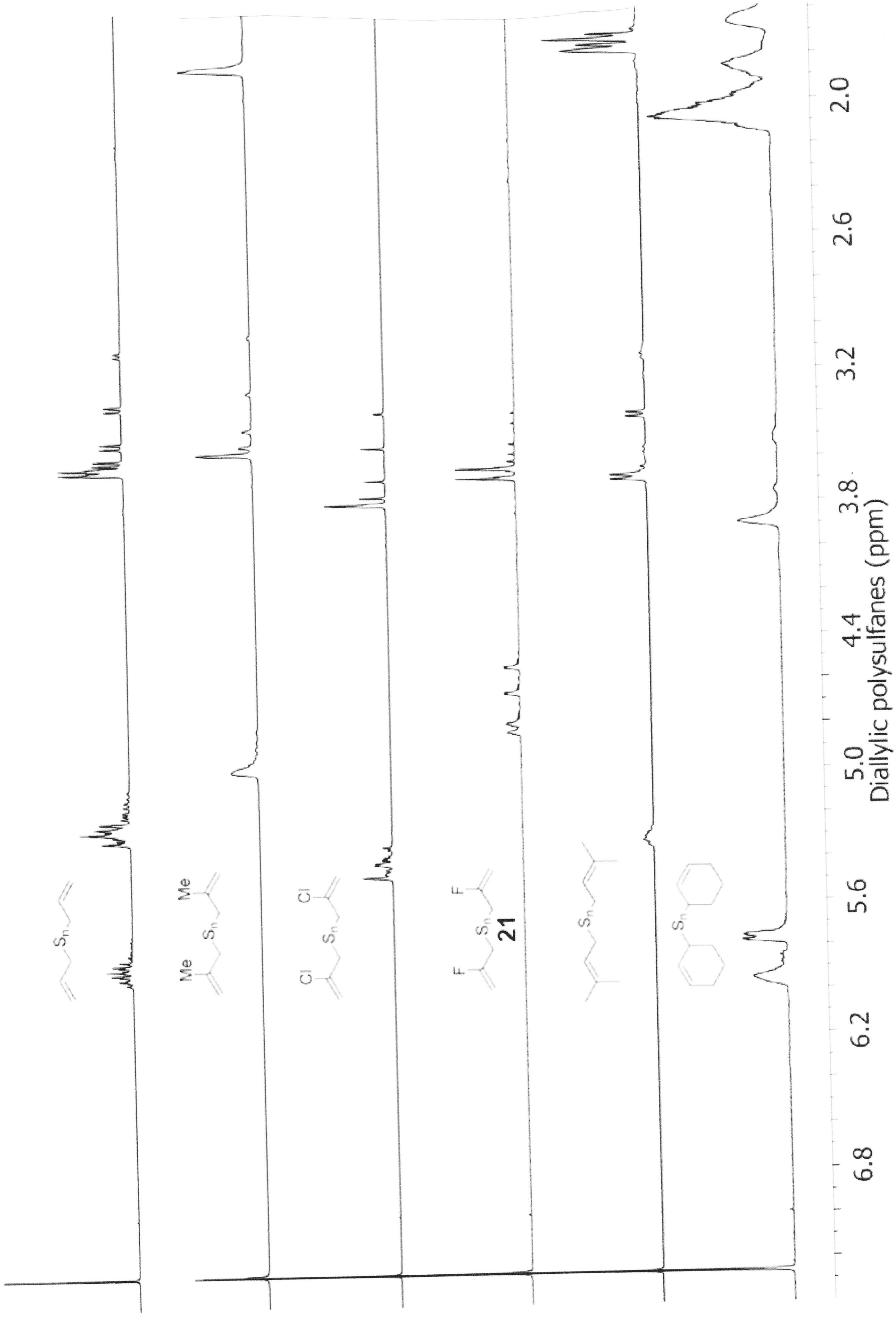

S-2 -Fluoro-2-propenyl-L-cysteine (**13**)

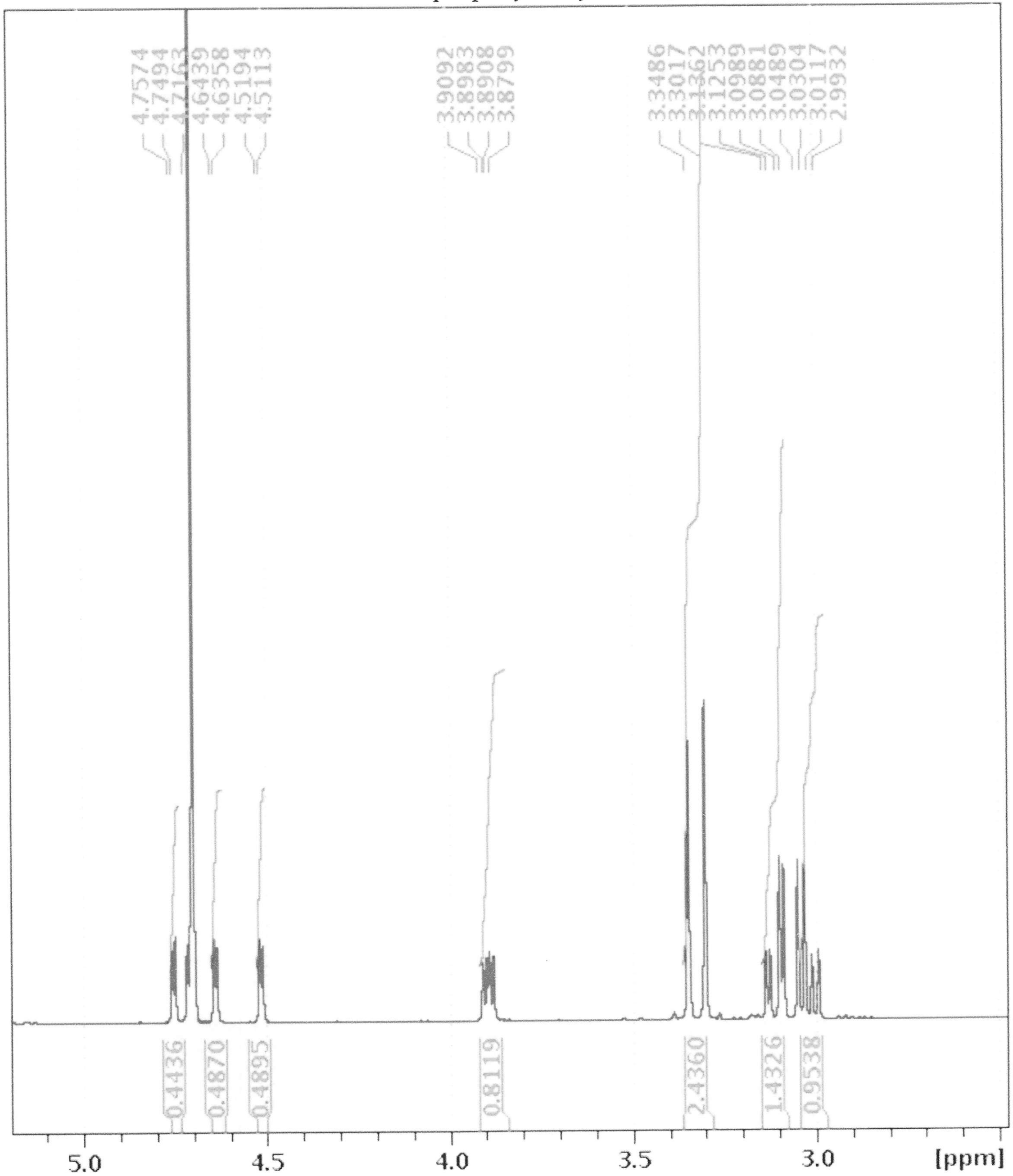

^{1}C NMR Spectra

Difluoroallicin (**12**; *S*-(2-Fluoro-2-propenyl) 2-fluoroprop-2-ene-sulfinothioate)

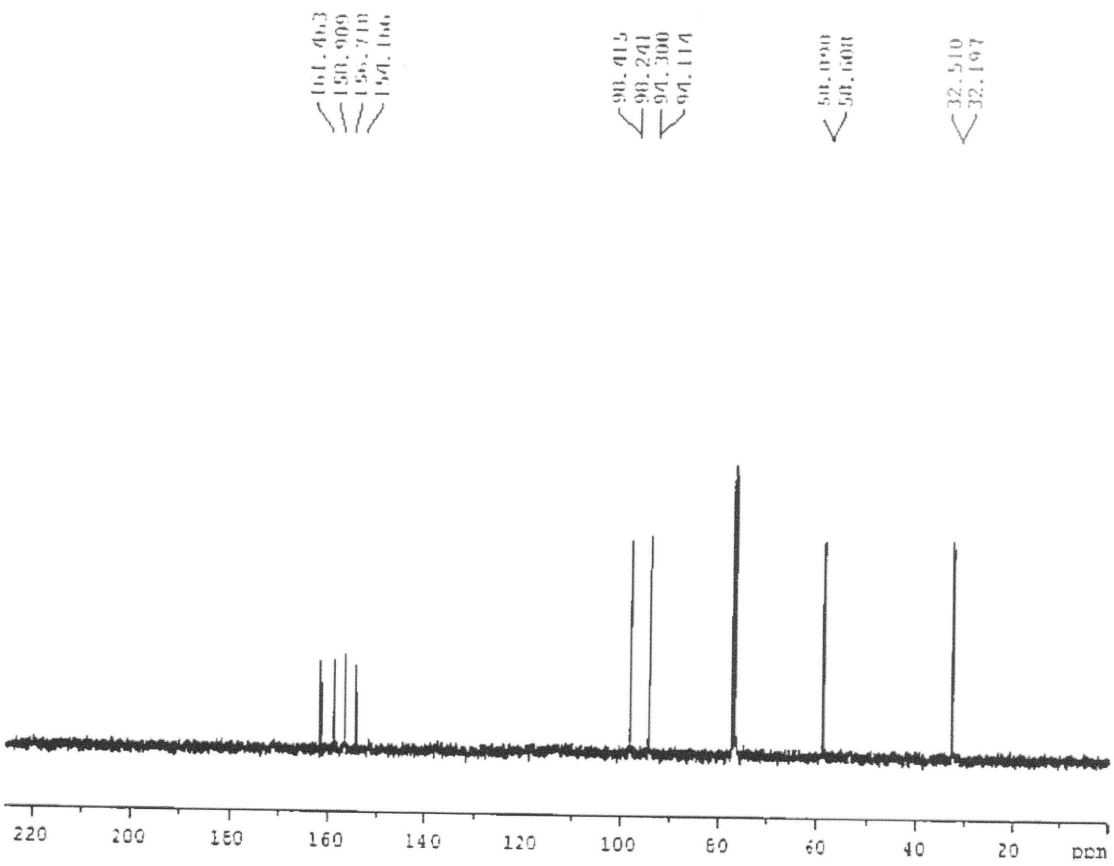

S-(2-Fluoro-2-propenyl)ethanethioate (**10**)

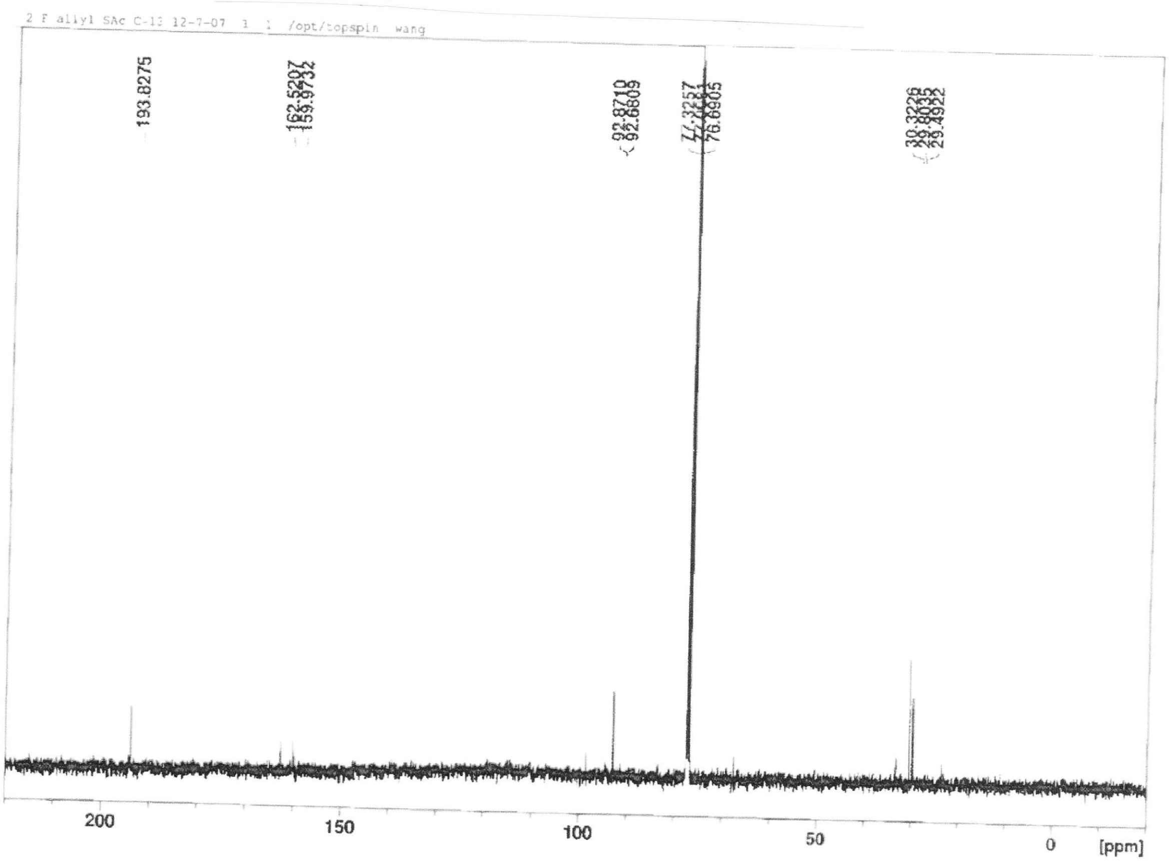

1,2- Bis(2-fluoro-2-propenyl)disulfane (**11**)

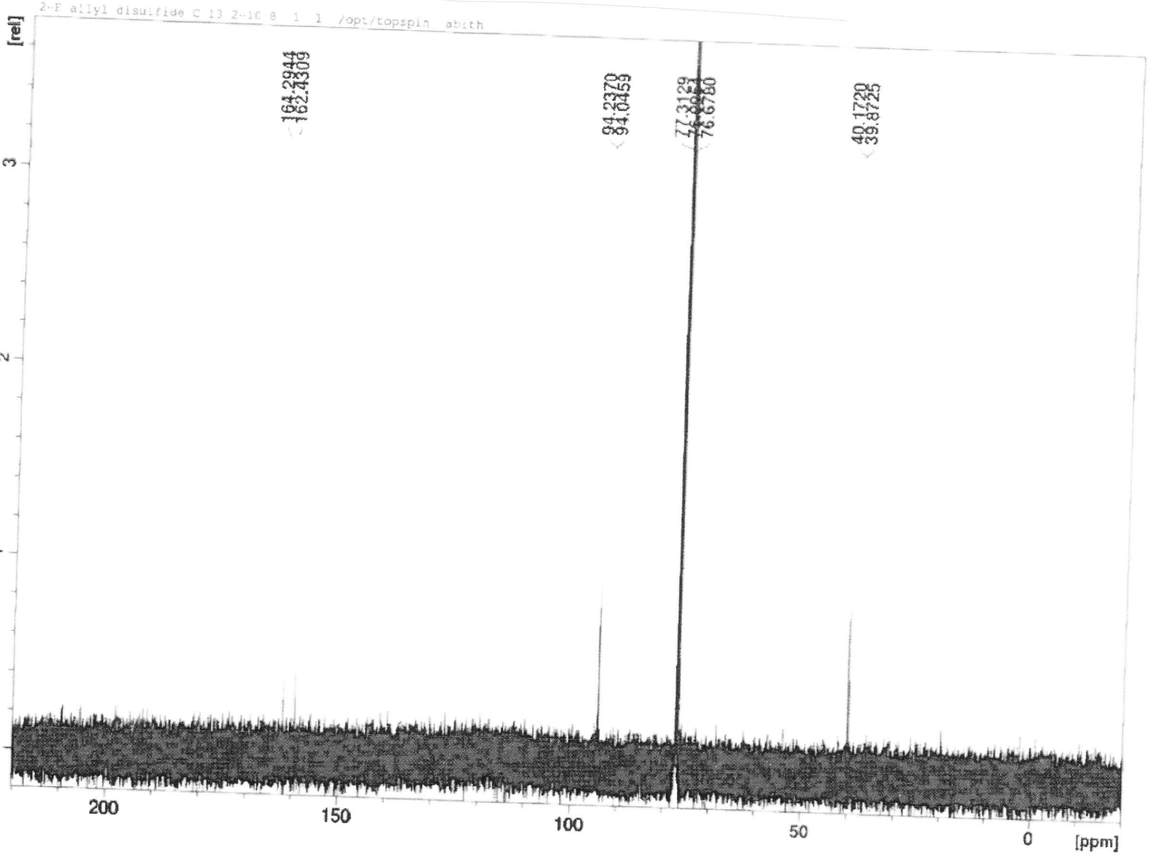

S-2 -Fluoro-2-propenyl-L-cysteine (**13**)

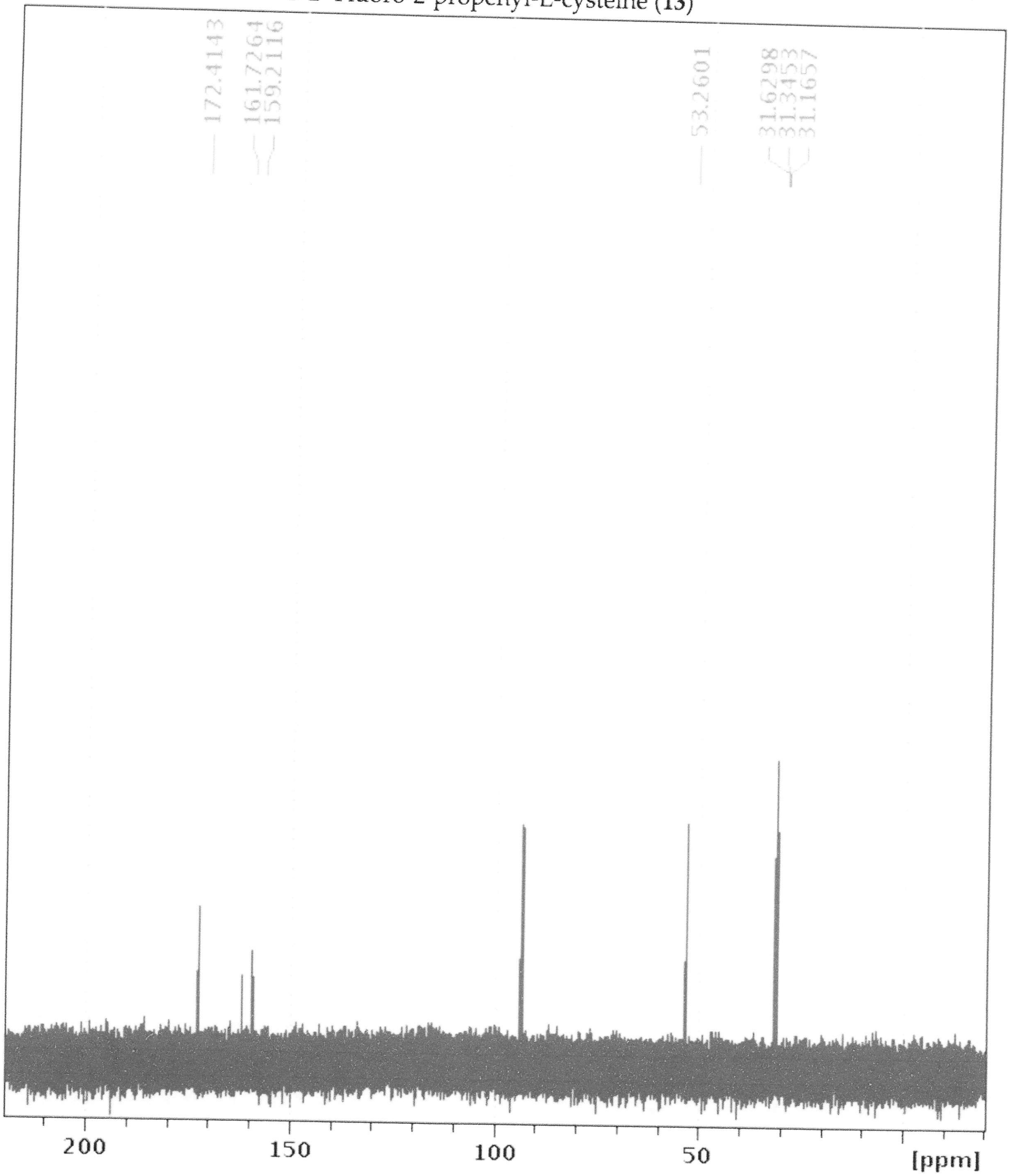

^{19}F NMR Spectra

Difluoroallicin (**12**; *S*-(2-Fluoro-2-propenyl) 2-fluoroprop-2-ene-sulfinothioate)

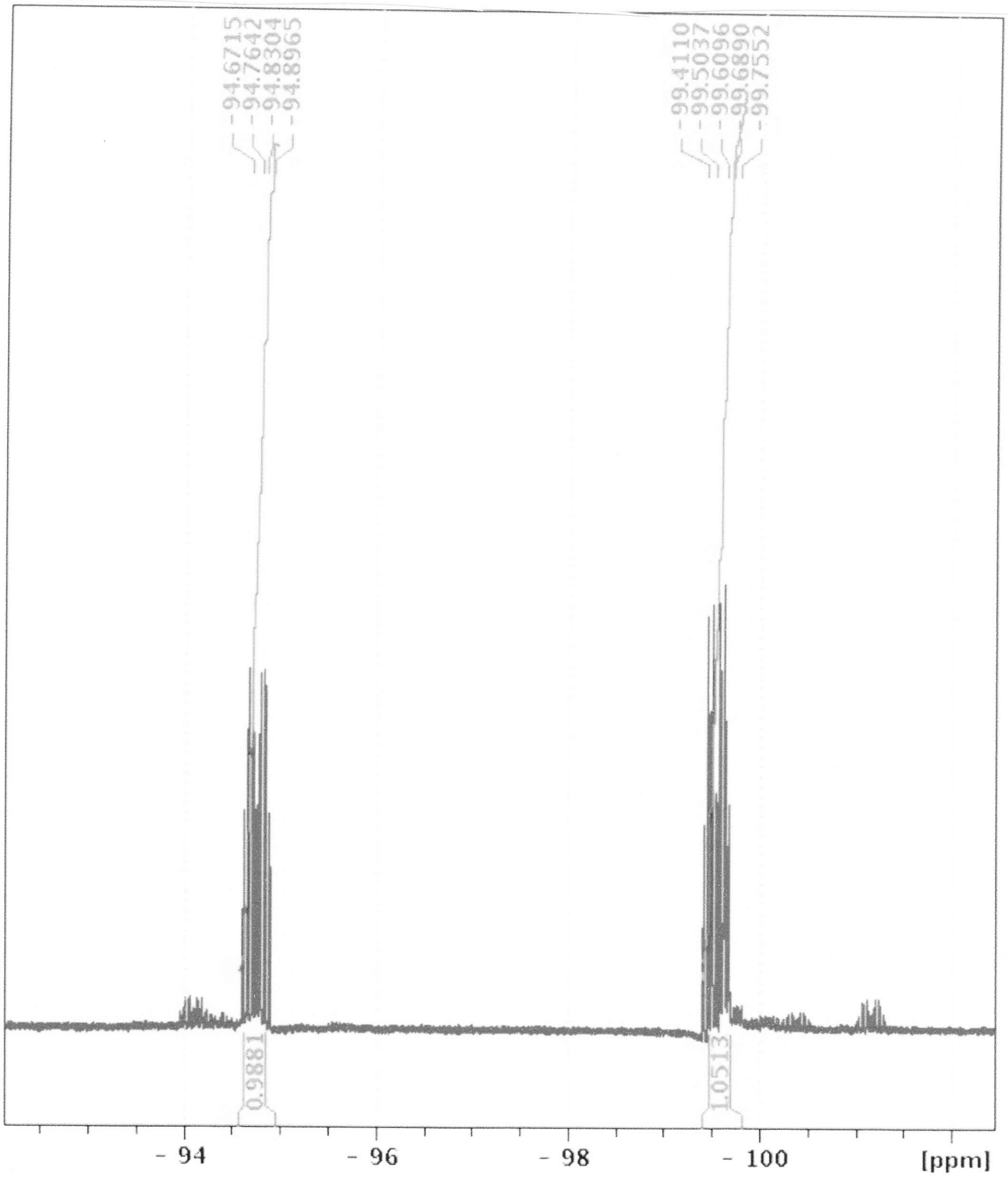

S-(2-Fluoro-2-propenyl)ethanethioate (**10**)

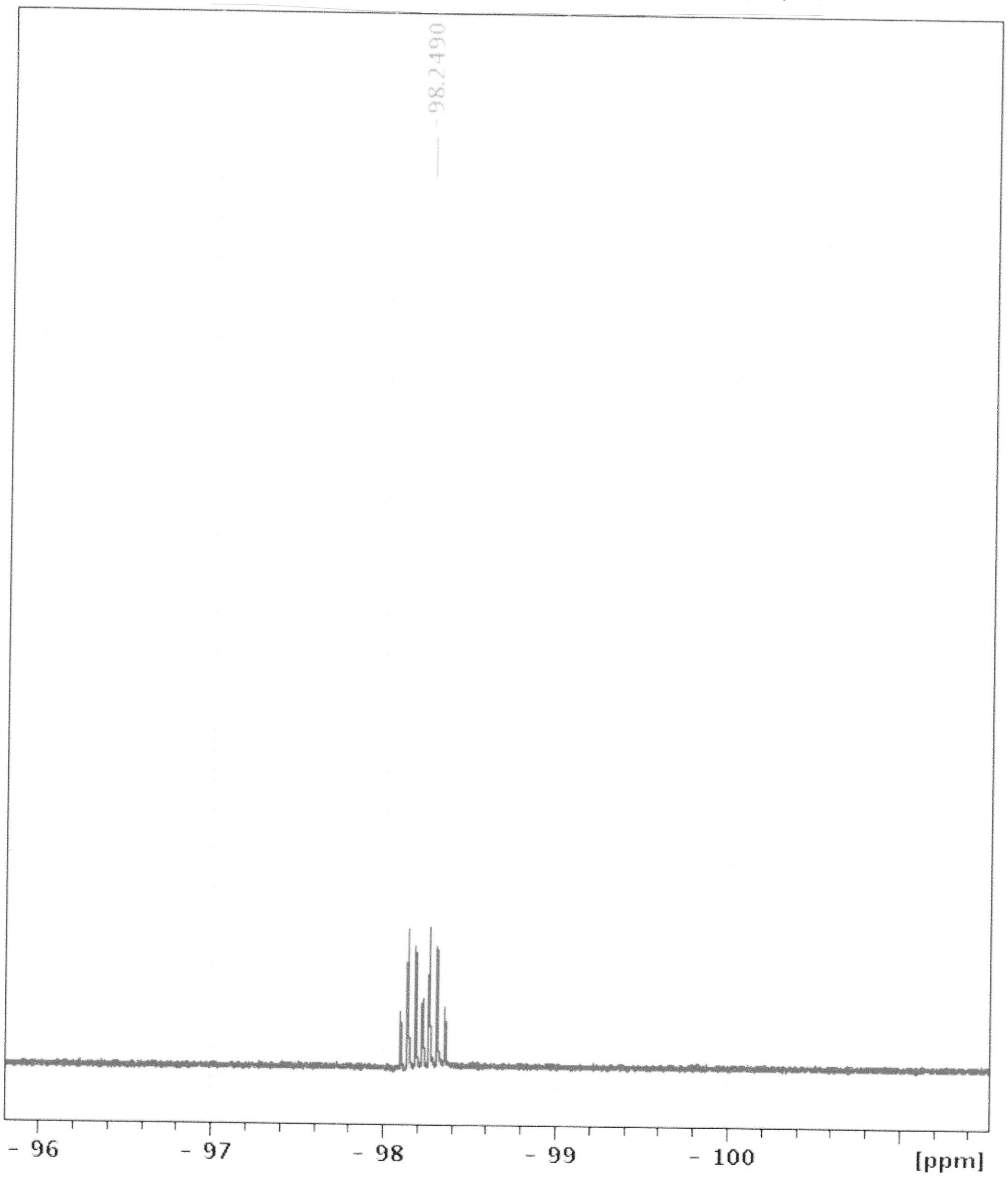

1,2- Bis(2-fluoro-2-propenyl)disulfane (**11**)

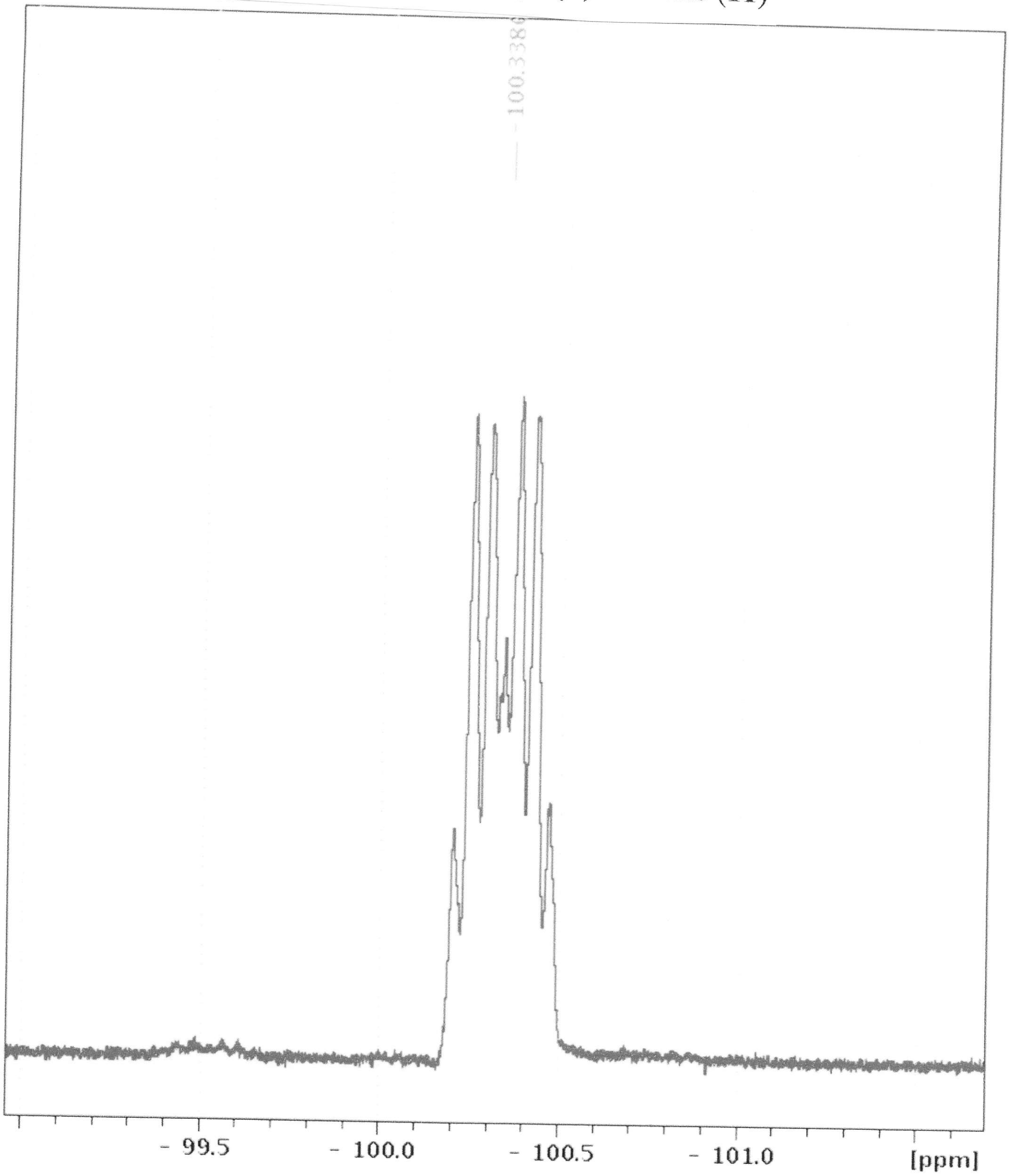

Mixture of difluoroallicin, **12**, and trifluoroajoene, **18**

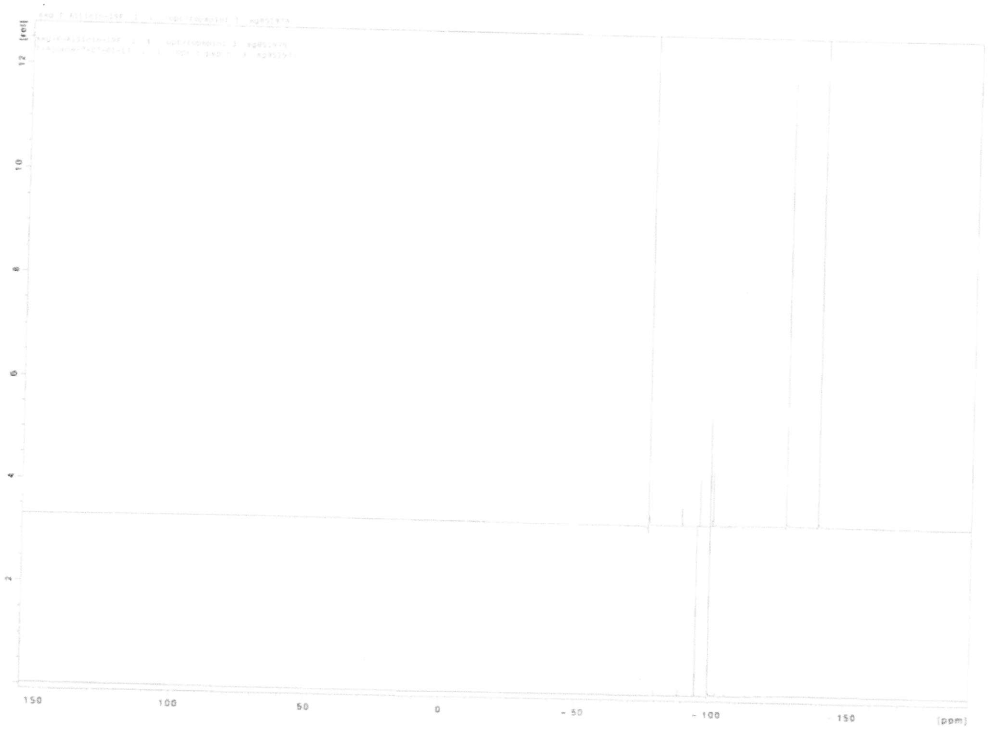

S-2 -Fluoro-2-propenyl-L-cysteine (**13**)

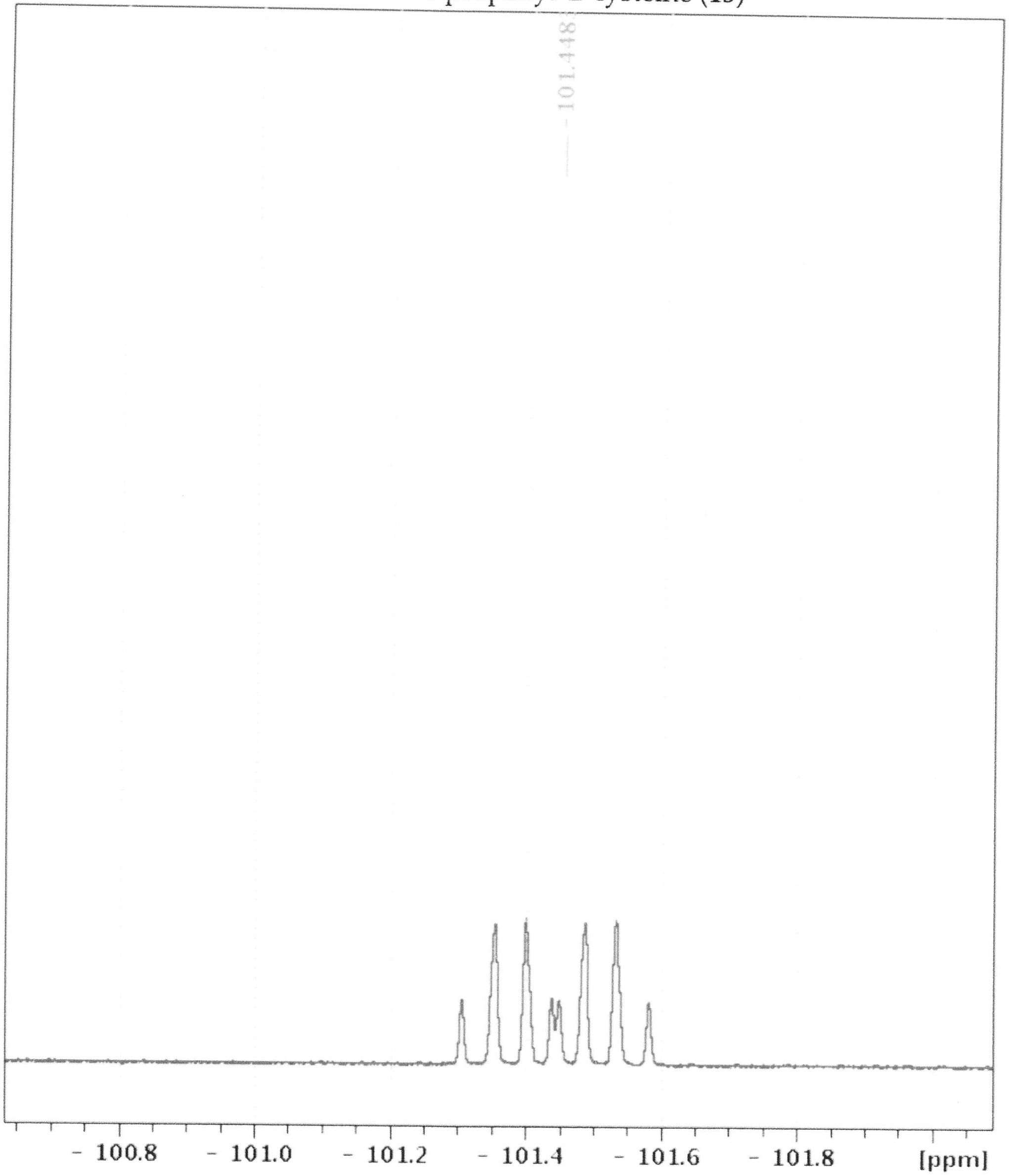

(+)-*S*-2-Fluoro-2-propenyl-L-cysteine *S*-oxide (**22**)

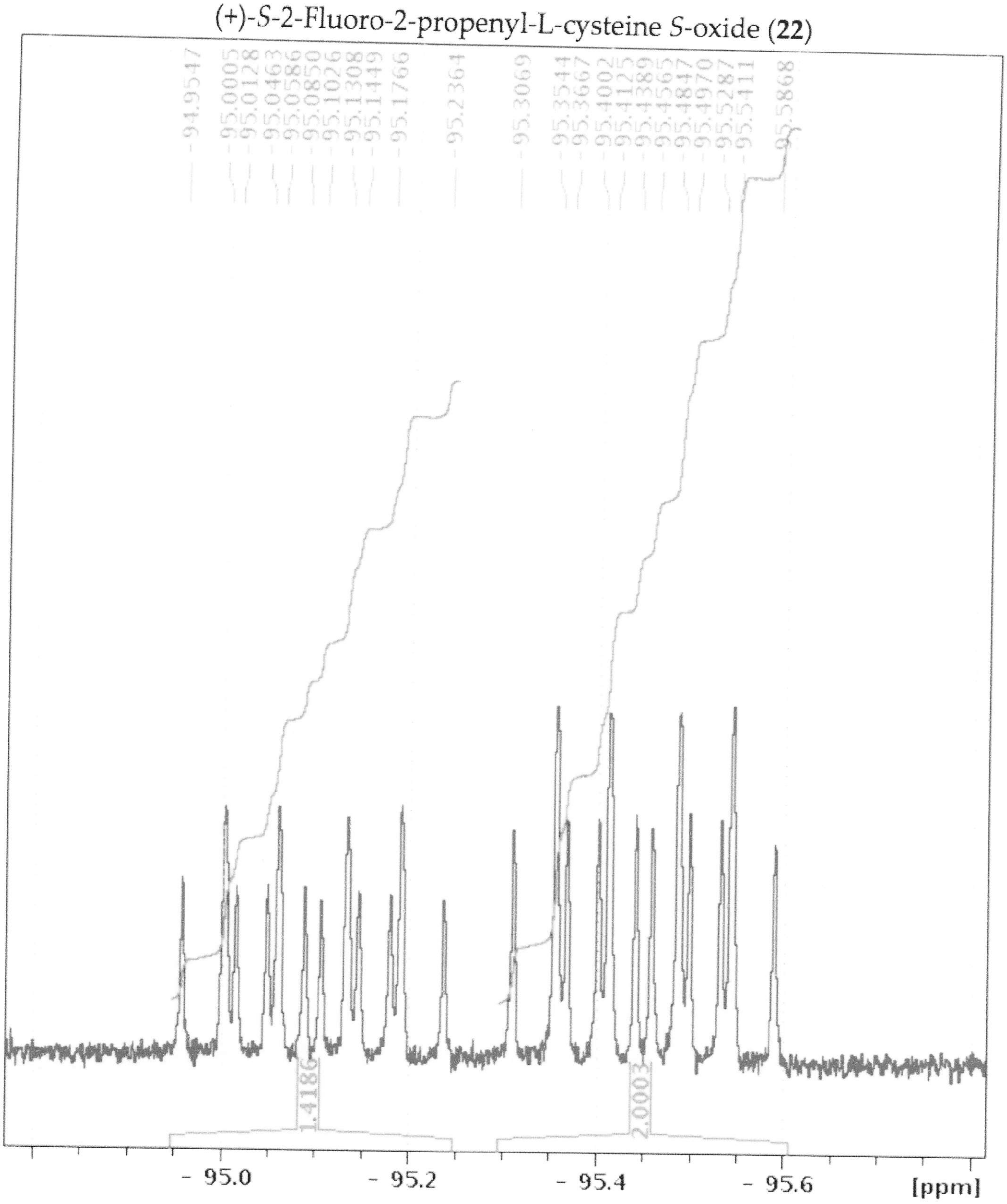

DART Mass Spectra

DART MS of fluoroalliin with powdered garlic showing allicin, monofluoroallicin and difluoroallicin [C6H9O1F2S2 (M+H) – diFluoroallicin Calc. m/z 199.0063 Found 199.0098; C6H10O1F1S2 (M+H)- monofluoroallicin? Calc. m/z 181.0157 Found 181.0169]

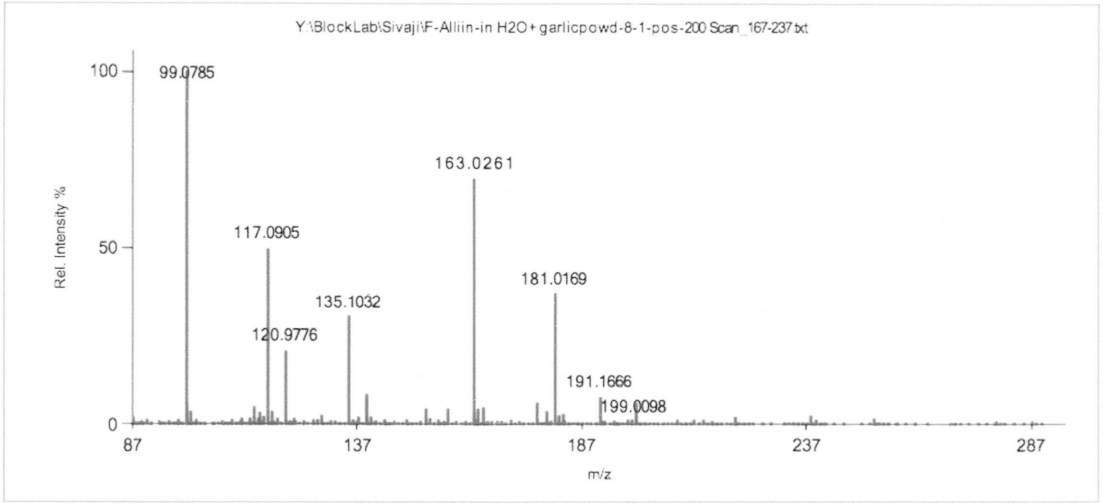

Mixture of difluoroallicin and trifluoroajoene

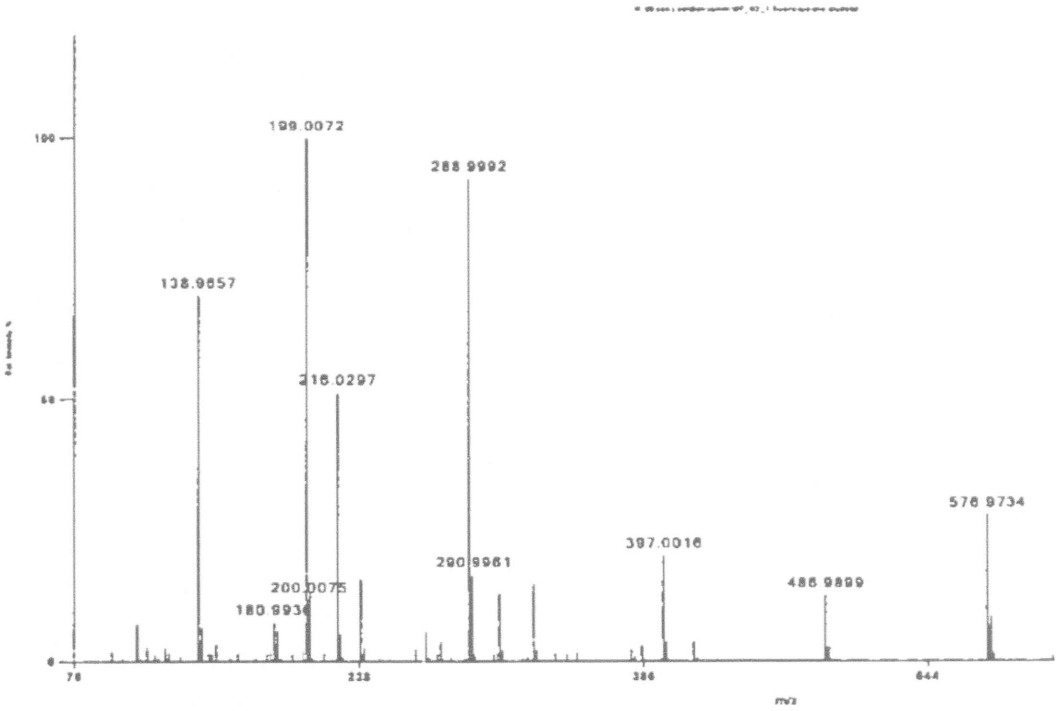

Supplement: Supplementary file 1 [file molecules-30-02841-s001.zip › molecules-3648220-supplementary.pdf]
